# Inactivation of Transcriptional Regulator FabT Influences Colony Phase Variation of Streptococcus pneumoniae

**DOI:** 10.1128/mBio.01304-21

**Published:** 2021-08-17

**Authors:** Jinghui Zhang, Weijie Ye, Kaifeng Wu, Shengnan Xiao, Yuqiang Zheng, Zhaoche Shu, Yibing Yin, Xuemei Zhang

**Affiliations:** a Key Laboratory of Diagnostic Medicine Designated by the Ministry of Education, Department of Laboratory Medicine, Chongqing Medical University, Chongqing, People’s Republic of China; b Department of Clinical Pharmacology, Xiangya Hospital, Central South University, Changsha, People’s Republic of China; c Department of Laboratory Medicine, The Third Affiliated Hospital of Zunyi Medical University (The First People’s Hospital of Zunyi), Zunyi, People’s Republic of China; d Department of Medicine Laboratory, Children’s Hospital of Chongqing Medical University Chongqing, People’s Republic of China; e Department of Blood Transfusion, The First Affiliated Hospital of Chongqing Medical University, Chongqing, People’s Republic of China; Yale University School of Medicine

**Keywords:** *Streptococcus pneumoniae*, capsular polysaccharide, phase variation, FabT, SpnD39III

## Abstract

Streptococcus pneumoniae is an opportunistic pathogen that can alter its cell surface phenotype in response to the host environment. We demonstrated that the transcriptional regulator FabT is an indirect regulator of capsular polysaccharide, an important virulence factor of Streptococcus pneumoniae. Transcriptome analysis between the wild-type D39s and D39Δ*fabT* mutant strains unexpectedly identified a differentially expressed gene encoding a site-specific recombinase, PsrA. PsrA catalyzes the inversion of 3 homologous *hsdS* genes in a type I restriction-modification (RM) system SpnD39III locus and is responsible for the reversible switch of phase variation. Our study demonstrated that upregulation of PsrA in a D39Δ*fabT* mutant correlated with an increased ratio of transparent (T) phase variants. Inactivation of the invertase PsrA led to uniform opaque (O) variants. Direct quantification of allelic variants of *hsdS* derivatives and inversions of inverted repeats indicated that the recombinase PsrA fully catalyzes the inversion mediated by IR1 and IR3, and FabT mediated the recombination of the *hsdS* alleles in PsrA-dependent and PsrA-independent manners. In addition, compared to D39s, the Δ*fabT* mutant exhibited reduced nasopharyngeal colonization and was more resistant to phagocytosis and less adhesive to epithelial cells. These results indicated that phase variation in the Δ*fabT* mutant also affects other cell surface components involved in host interactions.

## INTRODUCTION

S. pneumoniae is a significant human-pathogenic bacterium and resides asymptomatically in healthy carriers, and infections are primarily a consequence of decreased immunity in children and the elderly (1, 2). The primary outcome of S. pneumoniae infections is pneumonia that can be accompanied by invasive disease such as meningitis, sepsis, otitis media, and bacteremia (3, 4). The capsular polysaccharide (CPS) plays a critical role in virulence and forms a protective barrier from mucosal agglutination and opsonophagocytosis (5). The phenotypic variation of the amounts of capsule on the cell surface are driven by fluctuating environmental conditions in the host and are associated with the well-characterized and reversible phase variation characterized by colony opacity (opaque and transparent) (6–8). Phenotypic variants can be identified by examination of colony morphology (8).

Phenotypic diversity in bacterial populations is complex and involves a variety of intrastrain variations in cell surface features, and these include virulence proteins (9, 10), the capsule (7, 11–13), and pili (14, 15), as well as cell wall teichoic acids (16, 17). Phase variation is a very powerful contingency strategy for generating a diverse population that contains individual variants that allow the pneumococcus to adapt to survive specific host niches. For instance, the transparent variants which express a high level of teichoic acid and surface proteins are able to establish stable colonization on the mucosal surface in the nasopharyngeal mucosa (18). In contrast, the opaque variants which express more capsular polysaccharides and fewer teichoic acids are more resistant to host clearance and more virulent in sepsis models (6). Although the capsule is a significant pathogenic determinant, the molecular mechanisms underlying the relationship between the capsule and the phenotypic variation need further investigation.

Recent works have focused on an epigenetic mechanism controlling the reversible opaque/transparent phenotypic forms in S. pneumoniae (19–21). The phenotypic variation is controlled by a type I restriction modification (RM) system, SpnD39III, that consists of three cotranscribed genes, *hsdR* (restriction enzyme), *hsdM* (methyltransferase), and three homologous *hsdS* sequence recognition proteins. The latter are composed of an *hsdS* gene at the active site accompanied by two truncated and transcriptionally silent genes, *hsdS′* and *hsdS*″ (20). The *hsdS* gene consists of two variable regions encoding two target recognition domains (TRD), each of which identifies a specific sequence in the genome. The truncated *hsdS* homologs are responsible for providing additional alleles for both TRDs. Recombination between the *hsdS* genes is mediated by 3 pairs of inverted repeats, (IR) IR1 (85 bp), IR2 (298 bp), and IR3 (15 bp), that can recombine to form 6 unique *hsdS* alleles (19–23). The products of these alleles possess distinct DNA recognition specificities that generate distinct genomic DNA methylation patterns and thus control the phase variation of colony morphology. The DNA invertase PsrA is encoded within the SpnD39III locus and contributes to this site-specific DNA rearrangement (22, 23). This form of site-specific recombination is mediated by enzymes such as PsrA that catalyze the cleavage and rejoining of DNA fragments at specific recognition sequences without ATP or the synthesis of any new DNA. Indeed, SpnD39III TRD shuffling is not fully controlled by the site-specific recombinase PsrA. The inversion of a 15-bp IR3 bound sequence completely depends on PsrA, and the inversion within 85-bp IR1 and 298-bp IR2 bound sequences rely on factors in addition to PsrA (23), or for IR1 may be independent of PsrA (22). The regulation of *psrA* is unknown, and additional complexities are present in this system. For instance, the site-specific recombination can be mediated by direct repeats (19). The regulatory mechanisms underlying SpnD39III control of phase variation are far from clear.

Fatty acid biosynthesis transcriptional regulator FabT (SPD_0379) is a transcriptional repressor of membrane fatty acid synthesis in S. pneumoniae strains D39 and TIGR4 (24, 25). S. pneumoniae adopts the type II fatty acid synthesis pathway (FAS-II) for fatty acid synthesis, which is composed of 13 genes aligned in a single gene cluster, in which *fabM* encodes an enoyl-CoA hydratase/isomerase responsible for the synthesis of unsaturated fatty acids (UFAs) (25), whereas *fabK* encodes an enoyl-ACP reductase involved in saturated fatty acid synthesis (26). The promoter regions of *fabM*, *fabT*, and *fabK* contain a consensus palindromic sequence, which is under the control of FabT (24). A *fabT* knockout leads to an increased expression of all FAS-II genes with the exception of *fabM* (24, 25, 27) and decreases the production of UFA with shortened chain lengths (24). The fatty acid composition of cytoplasmic membrane was shown to be related to phase variation of S. pneumoniae (28). Compared to the transparent variants, the opaque variants possessed less UFA in the cell membrane (28). As suggested above, the function of transcriptional regulator FabT appears to have a close relationship with phase variation in S. pneumoniae.

In this study, we found that deletion of *fabT* in S. penumoniae strain D39 resulted in significant decrease in CPS production as well as colony phenotype alteration. Site-specific recombinase PsrA was upregulated in the *fabT*-deleted strain, which changed the IR-mediated inversions. In addition, alteration of *hsdS* allele composition was also found in the *fabT* mutant, though it was not fully dependent on IR-mediated inversions.

## RESULTS

### Deletion of *fabT* decreases capsular polysaccharide biosynthesis in S. pneumoniae D39.

A knockout of the *fabT* was problematic due to its cotranscription with the essential gene *fabH* (24). To prevent the downstream *fabH* from polar effects, we retained the 3′ 54 bp of *fabT* through the introduction of a Janus cassette 1 (19) by allelic exchange into a streptomycin-resistant D39 derivative strain (D39s). This resulted in the strain D39Δ*fabT*::JC1. The latter was then employed to generate an unmarked in-frame deletion of *fabT* designated strain D39Δ*fabT*. The genomic ectopic complement strain CΔ*fabT* was constructed by transformation of plasmid pPEPZ-Plac-RBS-*fabT*-FLAG3 encoding a Flag-tagged version of FabT. The absence of a *lacI* repressor in S. pneumoniae D39s ensured the constitutive expression of the construct. The *fabT* mRNA (Fig. S1A) and FabT protein (Fig. S1B) levels in this complementary strain were significantly lower than that in the parental strain D39s. These genomic manipulations did not result in any detectable differences in growth rates in Todd-Hewitt broth supplemented with yeast extract (THY) (data not shown).

10.1128/mBio.01304-21.1 FIG S1 Verification of *fabT* mutant strains. (A) Steady-state mRNA levels of *fabT* in *fabT* mutants; (B) FabT-Flag tag in complemented strain D39CΔ*fabT* detected by Western blotting. Download FIG S1, TIF file, 0.08 MB. Copyright © 2021 Zhang et al. 2021 Zhang et al. https://creativecommons.org/licenses/by/4.0/ This content is distributed under the terms of the Creative Commons Attribution 4.0 International license.

**FIG 1:**
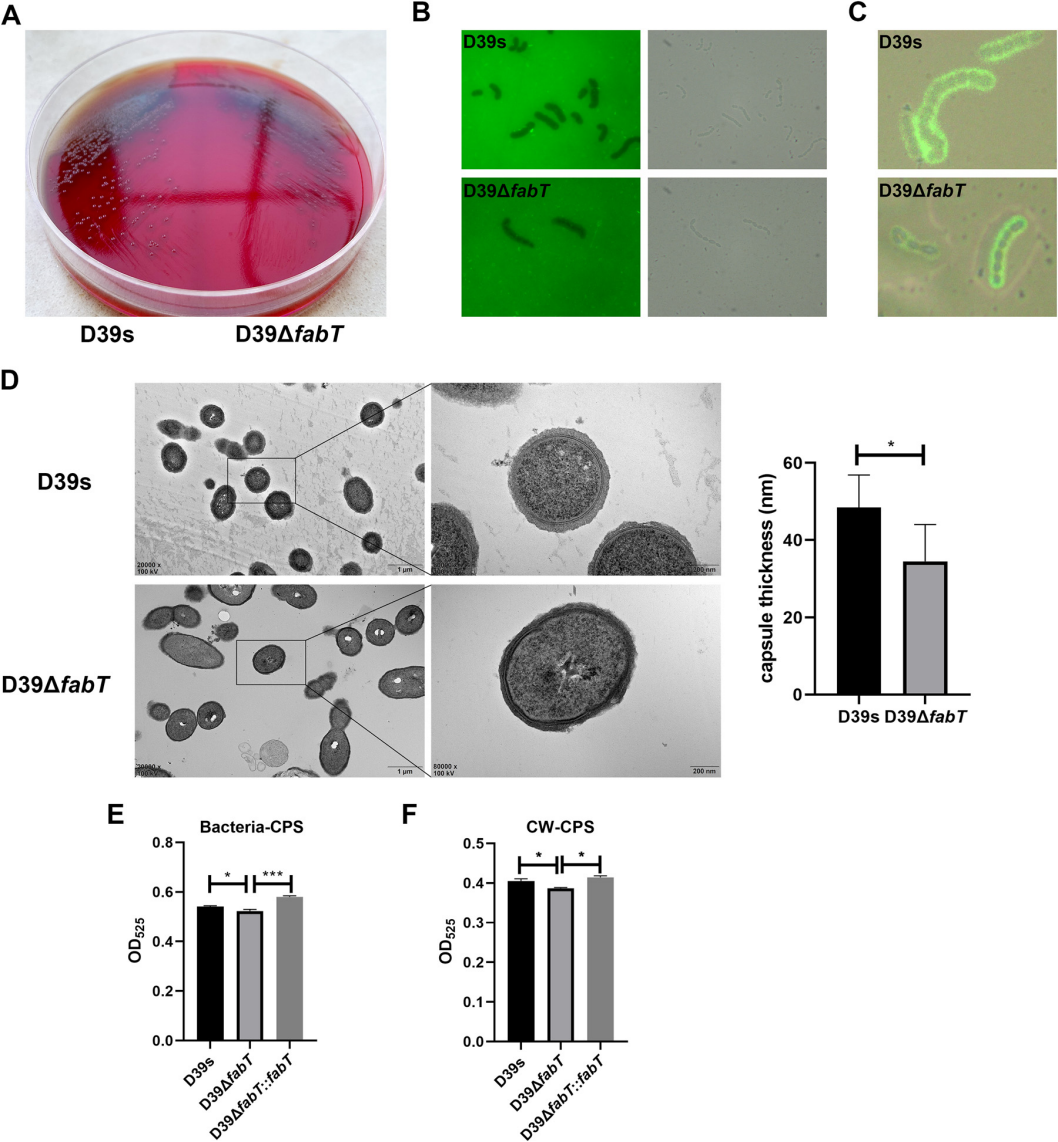
Deletion of *fabT* leads to decreased capsule production. (A) Colony morphology on a blood agar plate (BAP) of D39s (left) and D39Δ*fabT* (right) strains. (B) Fluorescence and bright-field microscopy of D39s (upper panel) and D39Δ*fabT* (lower panel) in the presence of FITC-dextran (×100 objective). (C) Overlay of bright-field and fluorescence microscopy of D39s and D39Δ*fabT* mutant strains showing the capsule in green (anti-type 2 capsule antibodies and FITC-goat anti-rabbit IgG). (D) Transmission electron microscopy of D39s and D39Δ*fabT.* The mean capsule layer diameters are indicated; *n* = 20. (E and F) Comparisons of whole-cell and cell wall-associated CPS using the uronic acid assay. Statistical analysis was performed using the unpaired *t test*. *, *P* < 0.05; **, *P* < 0.01; ***, *P* < 0.001.

The *fabT* gene knockout strain was composed of small and rough colonies on blood agar plates (Fig. 1A). This indicated that the Δ*fabT* mutant produced less CPS than the wild-type D39s (29, 30). We measured the capsular thickness of the Δ*fabT* mutant by exposing the bacteria to fluorescein isothiocyanate (FITC)-dextran, where the fluorescence-free area indicated the capsule boundary. The Δ*fabT* mutant displayed a smaller capsule size and a more transparent exclusion zone (Fig. 1B). Immunofluorescence microscopy revealed that the FabT-deficient strain showed a significant decrease in width of the fluorescence staining area with a smooth layer of capsule (Fig. 1C). This was verified by transmission electron microscopy (TEM), and the mean capsule layer diameter of the Δ*fabT* mutant was only 71.2% of that of the parental strain, which was 34.5 ± 9.5 nm, compared with 48.4 ± 8.5 nm for the parental D39s (Fig. 1D). CPS production was also assessed by measurements of uronic acid content among these strains. The uronic acid levels for the Δ*fabT* mutant were significantly less than those of the parental strain, and restoration of the gene in the complemented strain fully restored the uronic acid content, either for whole-cell CPS or the cell wall-associated CPS (Fig. 1E and F). These results indicated that deletion of the *fabT* gene substantially decreased CPS production as well as the CPS attached on the cell surface.

### Screening of FabT-regulated genes via transcriptomic analysis and qRT-PCR.

One potential mechanism for the capsule production defect in the Δ*fabT* mutant was downregulation of transcription at the *cps* locus. However, steady-state mRNA levels of the *cps* genes were unaffected by the *fabT* deletion (data not shown). Additionally, whole-genome transcriptome sequencing indicated that overall, 67 genes were upregulated and 66 were downregulated in the Δ*fabT* mutant (*P* < 0.05), and expression levels for the *cps* locus did not significantly vary between strains. As expected, the *fab* genes (SPD_0378-0390) were significantly increased from 1.7-fold to 4.4-fold in the Δ*fabT* mutant (Table 1). These results were inconsistent with previous studies (24, 27) in that *fabM* was also upregulated in the Δ*fabT* mutant, whereas this gene was previously shown to be independently regulated. The expression levels of *fabH* and *acpP* that are cotranscribed with *fabT* showed minor upregulation and no significant differences. This was supported by another study in which increased FabT expression resulted in a small decrease of *fabH* and did not affect *acpP* (31). These results were validated using a reverse transcription-quantitative PCR (qRT-PCR) analysis of the *fabM*, *fabT*, and *fabH* genes (Fig. S2A).

**TABLE 1:** Differential gene expression detected by transcriptome sequencing in the D39s relative to the D39Δ*fabT* mutant^*a*^

Regulation status in D39Δ*fabT* and gene no.^*b*^	Gene	Description	Log2 fold change	*P* value^*c*^
Up				
SPD_0140		ABC transporter ATP-binding protein	1.56	0.019
SPD_0185	*cls*	Cardiolipin synthase	0.72	0.008
SPD_0201	*rpmC*	50S ribosomal protein L29	0.65	0.046
SPD_0267		NCS2 family permease	0.63	0.012
SPD_0350	*vraT*	Cell wall-active antibiotic response protein	0.82	0.013
SPD_0351	*vraS*	Sensor histidine kinase	0.94	0.002
SPD_0354		DNA alkylation repair protein	1.16	0.032
SPD_0378	*fabM*	Enoyl-CoA hydratase	1.66	0.000
SPD_0380	*fabH*	Ketoacyl-ACP synthase III	0.73	0.000
SPD_0382	*fabK*	Enoyl-[acyl-carrier-protein] reductase FabK	2.05	0.000
SPD_0383	*fabD*	ACP S-malonyltransferase	1.77	0.000
SPD_0384	*fabG*	3-oxoacyl-[acyl-carrier-protein] reductase	1.70	0.000
SPD_0385	*fabF*	Beta-ketoacyl-ACP synthase II	1.76	0.000
SPD_0386	*accB*	Acetyl-CoA carboxylase biotin carboxyl carrier protein	1.79	0.000
SPD_0387	*fabZ*	3-Hydroxyacyl-ACP dehydratase FabZ	1.63	0.000
SPD_0388	*accC*	Acetyl-CoA carboxylase biotin carboxylase subunit	2.00	0.000
SPD_0389	*accD*	Acetyl-CoA carboxylase carboxyltransferase subunit beta	2.04	0.000
SPD_0390	*accA*	Acetyl-CoA carboxylase carboxyl transferase subunit alpha	2.13	0.000
SPD_0391	*briC*	Biofilm-regulating peptide BriC	1.19	0.004
SPD_0392		Hypothetical protein	0.87	0.031
SPD_0452	***psrA* ^***d***^**	Tyrosine-type DNA invertase PsrA	**3.03**	**0.000**
SPD_0453	***hsdS***	Restriction endonuclease subunit S	**2.93**	**0.000**
SPD_0501	*licT*	Transcription antiterminator Lict	1.95	0.000
SPD_0502		PTS glucose transporter subunit IIA	1.67	0.000
SPD_0503	*bglA-2*	6-Phospho-beta-glucosidase	1.69	0.000
SPD_0646		DegV family protein	1.49	0.000
SPD_0670		VanZ family protein	0.62	0.042
SPD_0671		ABC transporter ATP-binding protein	0.80	0.002
SPD_0684	*bioY*	Biotin transporter BioY	2.25	0.000
SPD_0688		ABC transporter permease	0.61	0.020
SPD_0691		PadR family transcriptional regulator	0.71	0.014
SPD_0703		DUF3270 domain-containing protein	1.31	0.020
SPD_0745	*plsY*	Glycerol-3-phosphate 1-O-acyltransferase PlsY	1.20	0.000
SPD_0772	*pfkB*	1-Phosphofructokinase	0.86	0.002
SPD_0858	*mutM*	DNA-formamidopyrimidine glycosylase	1.21	0.002
SPD_0859	*coaE*	Dephospho-CoA kinase	1.24	0.007
SPD_0860	*pmrA*	Multidrug efflux MFS transporter PmrA	1.16	0.019
SPD_0888	*adcAII*	Zinc-binding lipoprotein AdcAII	0.81	0.000
SPD_0889	*phtD*	Pneumococcal histidine triad protein PhtD	0.79	0.000
SPD_0890	*phtE*	Pneumococcal histidine triad protein PhtE	1.51	0.000
SPD_0913		DUF1002 domain-containing protein	1.77	0.014
SPD_0916	*piaB*	Iron ABC transporter permease	1.39	0.003
SPD_0917	*piaC*	Iron ABC transporter permease	1.38	0.008
SPD_0918	*piaD*	ABC transporter ATP-binding protein	1.20	0.015
SPD_1037	*phtB*	Pneumococcal-type histidine triad protein	0.69	0.008
SPD_1049	*lacT*	Transcription antiterminator	1.92	0.016
SPD_1164	*cdd-2*	Cytidine deaminase	2.18	0.000
SPD_1165		Phosphatidylglycerophosphatase A	1.30	0.000
SPD_1166		Hypothetical protein	1.34	0.000
SPD_1167	*appD*	ABC transporter ATP-binding protein	1.43	0.000
SPD_1168	*appC*	ABC transporter permease	1.54	0.000
SPD_1169	*appB*	ABC transporter permease	1.54	0.000
SPD_1170	*appA*	ABC transporter substrate-binding protein	1.16	0.007
SPD_1171		Cyclically permuted mutarotase family protein	1.30	0.000
SPD_1264		ABC transporter ATP-binding protein	1.35	0.015
SPD_1266		Energy-coupling factor transporter transmembrane protein EcfT	1.48	0.016
SPD_1267		ABC transporter ATP-binding protein	1.32	0.003
SPD_1276		EamA family transporter	0.91	0.004
SPD_1277		Serine hydrolase	0.79	0.013
SPD_1490		YesL family protein	1.29	0.016
SPD_1492	*yjgK*	Protein YjgK, linked to biofilm formation	1.71	0.003
SPD_1628	*xpt*	Xanthine phosphoribosyltransferase	1.11	0.003
SPD_1629	*pbuX*	Xanthine permease	1.13	0.001
SPD_2043	*pcsB*	Secreted antigen GbpB/SagA/PcsB, putative peptidoglycan hydrolase	1.41	0.033
SPD_2068	*htrA*	Serine protease, DegP/HtrA	1.48	0.012
Down				
SPD_0004	*ychF*	Redox-regulated ATPase YchF	–0.59	0.037
SPD_0024	*purA*	Adenylosuccinate synthase	–0.55	0.019
SPD_0033	*prsA*	Ribose-phosphate diphosphokinase	–0.84	0.002
SPD_0063	*strH*	Beta-N-acetylhexosaminidase	–0.67	0.037
SPD_0074		Nucleoside phosphorylase	–0.74	0.015
SPD_0079		Hypothetical protein	–0.79	0.022
SPD_0098		Glycosyltransferase family 2 protein	–0.63	0.009
SPD_0340	*rnpB*	RNase P RNA component class B	–0.66	0.003
SPD_0369	*zapA*	Cell division protein ZapA	–0.68	0.010
SPD_0373	*ahpD*	Alkyl hydroperoxide reductase AhpD	–1.55	0.000
SPD_0379	*fabT*	MarR family transcriptional regulator	–15.50	0.000
SPD_0422		Hypothetical protein	–0.98	0.005
SPD_0423	*xylR*	Xylose repressor protein	–0.69	0.008
**SPD_0450**	***hsdS***	**Restriction endonuclease subunit S**	–**3.07**	**0.000**
SPD_0495		DUF3883 domain-containing protein	–**0.74**	0.032
SPD_0534	*estA*	Esterase family protein	–0.66	0.005
SPD_0558	*prtA*	Serine protease PrtA precursor	–1.19	0.005
SPD_0582		DUF3042 family protein	–0.99	0.032
SPD_0610		Hypothetical protein	–2.67	0.000
SPD_0611		Hypothetical protein	–2.17	0.002
SPD_0612		Hypothetical protein	–1.94	0.001
SPD_0613		Hypothetical protein	–1.64	0.001
SPD_0614		ABC transporter ATP-binding protein	–1.71	0.000
SPD_0637		lactoylglutathione lyase	–1.21	0.005
SPD_0649	*upp*	Uracil phosphoribosyltransferase	–0.96	0.000
SPD_0700	*pepN*	M1 family metallopeptidase	–0.59	0.034
SPD_0718		YkuJ family protein	–1.13	0.002
SPD_0754		DUF2969 domain-containing protein	–0.72	0.039
SPD_0853	*lytB*	Endo-beta-*N*-acetylglucosaminidase	–2.67	0.000
SPD_0957	*dnaG*	DNA primase	–0.81	0.000
SPD_0958	*rpoD*	RNA polymerase sigma factor RpoD	–0.69	0.001
SPD_0959		Metal-sulfur cluster assembly factor	–0.85	0.012
SPD_0997	*hup*	HU family DNA-binding protein	–0.87	0.003
SPD_1141	*uraA*	Uracil transporter	–2.52	0.002
SPD_1212	*ywbD*	Putative ribosomal RNA large subunit methyltransferase YwbD	–0.58	0.033
SPD_1274	*guaA*	Glutamine-hydrolyzing GMP synthase	–0.53	0.032
SPD_1295		Hemolysin III family protein	–0.80	0.022
SPD_1431	*gtrB*	Bactoprenol glucosyl transferase	–0.80	0.007
SPD_1461	*psaB*	Manganese ABC transporter, ATP-binding protein	–1.98	0.009
SPD_1462	*psaC*	Manganese ABC transporter, permease protein, putative	–1.97	0.006
SPD_1463	*psaA*	Metal ABC transporter substrate-binding lipoprotein/adhesin PsaA	–1.88	0.022
SPD_1468	*gpmA*	Phosphoglycerate mutase	–0.98	0.001
SPD_1483	*murF*	UDP-*N*-acetylmuramoyl-tripeptide–d-alanyl-d-alanine ligase	–0.78	0.000
SPD_1548	*gmk*	Guanylate kinase	–0.48	0.042
SPD_1581		tRNA-Lys-CUU	–1.11	0.000
SPD_1594		XRE family transcriptional regulator	–0.69	0.009
SPD_1595		Hypothetical protein	–0.76	0.014
SPD_1649	*piuB*	Iron-compound ABC transporter, Permease protein	–2.13	0.011
SPD_1650	*piuC*	Iron-compound ABC transporter, permease protein	–4.54	0.000
SPD_1651	*piuD*	Iron-compound ABC transporter, ATP-binding protein	–2.98	0.000
SPD_1652	*piuA*	Iron-compound ABC transporter, iron-compound-binding protein	–2.88	0.018
SPD_1775	*lysA*	Diaminopimelate decarboxylase	–0.71	0.024
SPD_1790	*rpmH*	50S ribosomal protein L34	–0.82	0.048
SPD_1899		Gamma-glutamyl-gamma-aminobutyrate hydrolase family protein	–1.35	0.022
SPD_1903	*mutS*	DNA mismatch repair protein MutS	–0.54	0.041
SPD_1922	*hipO*	*N*-acetyldiaminopimelate deacetylase	–0.59	0.043
SPD_1923	*dapD*	2,3,4,5-Tetrahydropyridine-2,6-dicarboxylate *N*-acetyltransferase	–0.85	0.004
SPD_1926	*tyrS*	Tyrosine-tRNA ligase	–1.07	0.002
SPD_1927	*ctpC*	Heavy metal translocating P-type ATPase	–0.96	0.000
SPD_1931		Membrane protein	–0.74	0.020
SPD_1984	*ybbK*	Putative stomatin/prohibitin-family membrane protease subunit YbbK	–1.09	0.003
SPD_2018		Isoprenylcysteine carboxyl methyltransferase family protein	–0.80	0.033
SPD_2055	*guaB*	IMP dehydrogenase	–0.86	0.003

a Log2 fold change in gene expression as assessed by RNA-seq.

b The reference genome under GenBank accession numbers NC_008533.2 and CP027540.1.

c Adjusted *P* values, *P* values were adjusted using the Benjamini and Hochberg method.

d SpnD39III type I restriction-modification system genes are indicated in bold.

10.1128/mBio.01304-21.2 FIG S2 (A to D) The qRT-PCR results for the (A) *fab* gene cluster (B) *adcAII* (C) *hsdM*, and (D) *mutS* in the D39s and D39s derivative strains. Download FIG S2, TIF file, 0.1 MB. Copyright © 2021 Zhang et al. 2021 Zhang et al. https://creativecommons.org/licenses/by/4.0/ This content is distributed under the terms of the Creative Commons Attribution 4.0 International license.

The zinc transporter lipoprotein AdcAII was recently found to affect capsule thickness (32) and was upregulated 1.75-fold in Δ*fabT* mutants as detected using transcriptome analysis. However, the quantitative RT-PCR analysis did not reproduce this difference in our study (Fig. S2B). We also found that a variety of membrane-associated ABC transporters were altered in the transcriptome analysis. These included iron-ABC transporters PiaABC (SPD_0916-0918) and piuABC (SPD_1650-1652), manganese-ABC transporter PsaABC (SPD_1641-1643), and oligopeptide ABC transporter (SPD_1168-1170) (33–35). These alterations were most likely the result of the overall cellular response to the changes in membrane fatty acid composition as previously reported (24, 36).

**FIG 2:**
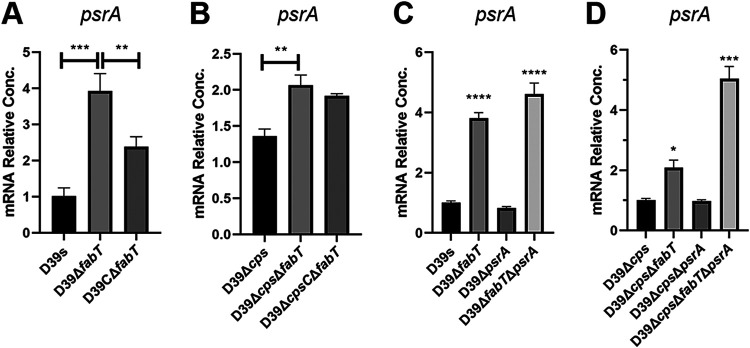
*psrA* gene expression in *fabT* and *psrA* mutant strains. (A to D) Related abundance of the *psrA* mRNA in the (A and C) encapsulated and (B and D) unencapsulated *fabT* and *psrA* mutant strains was detected by qRT-PCR. Results were normalized to *gyrB*. Data are shown as the mean ± SEM of a representative experiment. Each experiment was replicated at least three times. Statistical analysis was performed using the unpaired *t* test. *, *P* < 0.05; **, *P* < 0.01; ***, *P* < 0.001; ****, *P* < 0.0005.

Of particular interest, the Δ*fabT* mutant also displayed a significant upregulation of *psrA* as well as *hsdS′* and an associated downregulation of *hsdS*. The transcriptional upregulation of *psrA* was validated by qRT-PCR and was less obvious in the unencapsulated Δ*cps*Δ*fabT* strain. The complemented FabT strain displayed *psrA* levels that were partially reduced (Fig. 2A and B).

To further confirm the linkage between FabT and PsrA, we constructed frameshift *psrA* mutants in strains D39s and D39Δ*cps* by truncating the PsrA protein at amino acid 6 without altering the size of the gene, and mRNA levels did not vary even though no functional PsrA was expressed. The mutation of *psrA* in the Δ*fabT* mutant resulted in high-level *psrA* expression in both encapsulated and unencapsulated strains (Fig. 2C and D). This result indicated that FabT might participate in the negative feedback regulation of PsrA that inhibited its own expression when FabT was present.

### Deletion of *fabT* drives pneumococci toward the T phase by the PsrA-dependent mechanism.

**FIG 3:**
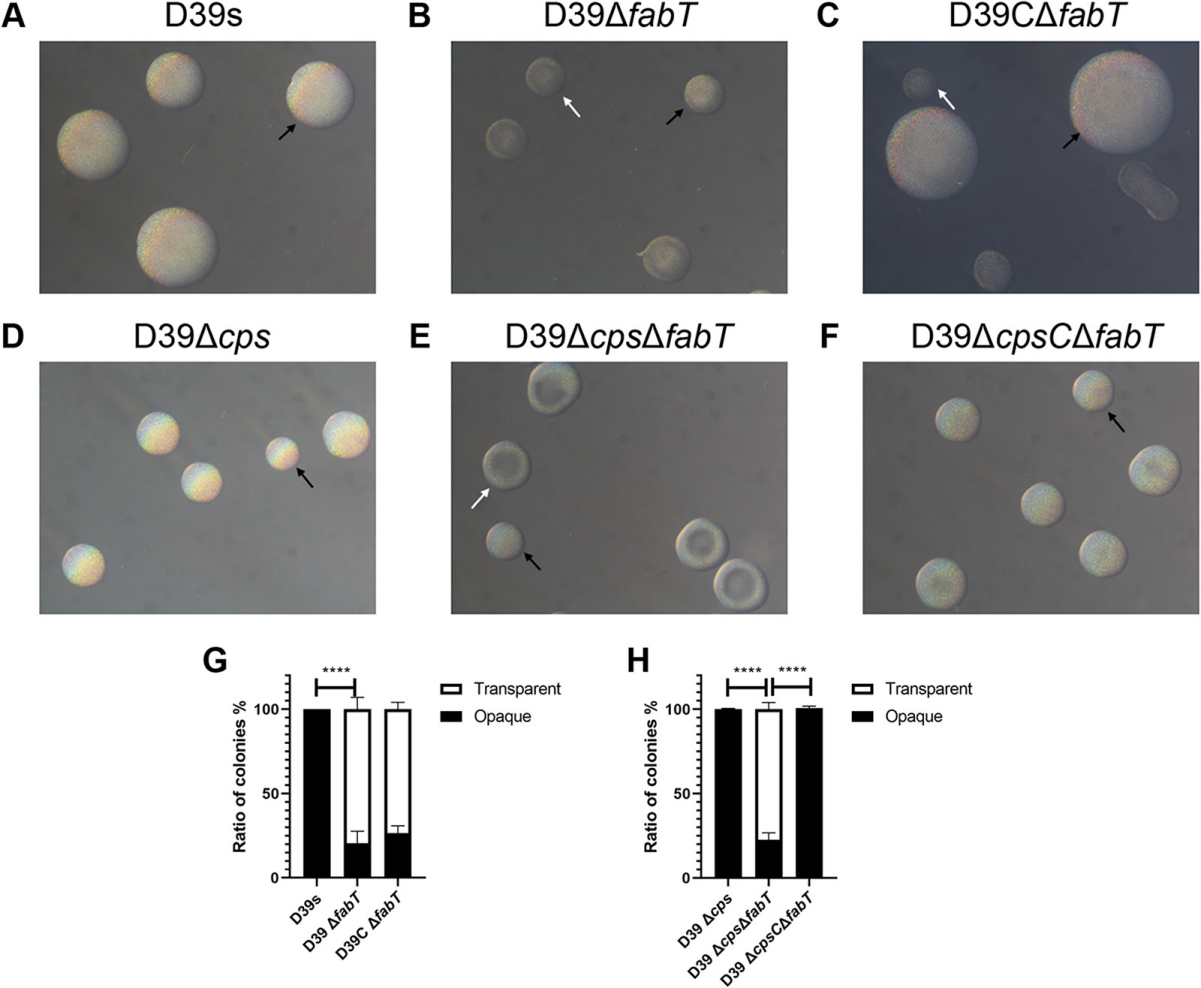
Colony morphology of *fabT* mutants. (A to C) Encapsulated D39 and derivatives and (D to F) unencapsulated D39Δ*cps* and derivatives were grown on TSA plates supplemented with catalase. Phase variation of colony opacity of S. pneumoniae was visualized under an inverted microscope through oblique lighting after 24 h (encapsulated) and 26 h (unencapsulated). Representative opaque (O) and transparent (T) colonies in each strain are highlighted with black and white arrowheads, respectively. (G) O/T ratio of encapsulated strains. (H) O/T ratio of unencapsulated strains. All pictures are to the same scale. Original magnification  = ×40.

S. pneumoniae colonies can switch between transparent (T) and opaque (O) phenotypes known as phase variation. These variants differ in expression of surface virulence proteins, CPS, and teichoic acids. We examined whether FabT regulates CPS production through phase variation. We first investigated whether the differentially expressed *psrA* and *hsdS* in the Δ*fabT* mutant affect phase variation. The colony phenotypes and the ratio between O and T colonies in a single clonally derived population were characterized for D39s and the Δ*fabT* mutant on tryptic soy agar (TSA) plates. Parental strain D39s was uniformly composed of O colonies (100% O) after 24 h. In contrast, *fabT* deletion led to a significantly increased proportion of T colonies compared with the parental strain (79.3% T). Interestingly, the D39CΔ*fabT* strain generated two colony types. The shape and size of large colonies were similar to those of D39s and were uniform and opaque and accompanied by small colonies that were predominantly the T phenotype (Fig. 3C). Moreover, these distinct variants in D39CΔ*fabT* were unable to switch phases with extensive passaging (Table S1). Mechanisms of adaptation leading to pneumococcal irreversible mutations and to readjustment of regulatory networks have been described (37). The complemented D39CΔ*fabT* strain was present as two stable and heritable variant colonies that may be related to this type of readjustment process.

10.1128/mBio.01304-21.7 TABLE S1 Quantitative assessment of the stability in the colony opacity phenotypes among the D39s derivatives. Download Table S1, PDF file, 0.05 MB. Copyright © 2021 Zhang et al. 2021 Zhang et al. https://creativecommons.org/licenses/by/4.0/ This content is distributed under the terms of the Creative Commons Attribution 4.0 International license.

Previous reports have indicated that the thick capsule in D39 derivatives may blur the observation of colony opacity, resulting in an incorrect interpretation of the O/T phenotype (19). To eliminate the potential for this in our experiments, we generated unencapsulated D39Δ*cps* and Δ*cps*Δ*fabT* strains and then evaluated colony phenotypes. Though it is typically assumed that unencapsulated pneumococci undergo spontaneous phase variation in colony opacity, unencapsulated D39Δ*cps* still produced a uniformly opaque colony after 26 h of cultivation (100% O). In contrast, the Δ*cps*Δ*fabT* mutant presented a mixture at 26 h (77.3% T) (Fig. 3D and E). The colony morphology of unencapsulated CΔ*fabT* reverted to a uniformly opaque variant (Fig. 3F). These results indicated that FabT regulated the phase variation in S. pneumoniae independently of CPS expression.

**FIG 4:**
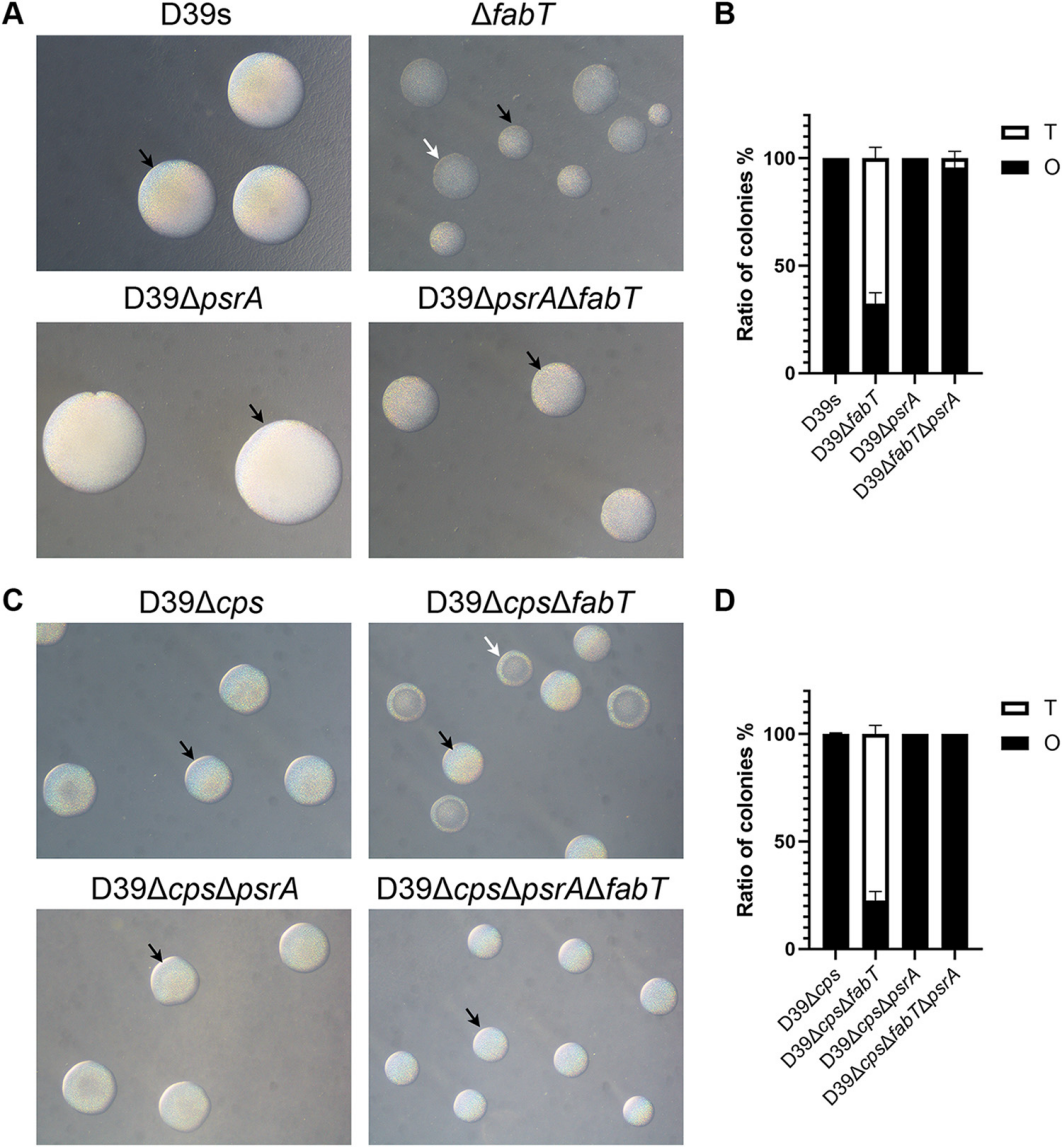
Colony morphology of *fabT* and *psrA* mutants. (A) Encapsulated D39 and derivatives and (C) unencapsulated D39Δ*cps* and derivatives cultured on TSA supplemented with catalase. Phase variation was visualized through oblique lighting after 24 h (encapsulated) and 26 h (unencapsulated). (B) O/T ratio of encapsulated strains. (D) O/T ratio of unencapsulated strains. See Fig. 3 for abbreviations and additional information.

To further determine whether the variation of colony morphology in the Δ*fabT* mutant was due to the upregulated PsrA, we determined the phase variation in inactivated *psrA* mutants. The *psrA* mutants that were generated from Δ*fabT* mutants produced almost completely opaque colonies (Fig. 4). These results indicated that FabT was able to regulate colony opacity in a PsrA-dependent manner. However, complemented Δ*cps*CΔ*fabT* did not downregulate the expression of psrA significantly, indicating that FabT was also able to regulate colony opacity in a PsrA-independent manner.

Interestingly, the capsule produced by Δ*psrA* mutants was not significantly different from its parental strain (Fig. S3). In addition, the unencapsulated *fabT* and *psrA* mutants showed consistent colony opacity variation, indicating that lower levels of expressed capsule in the Δ*fabT* mutant were not associated with increased numbers of T variants (Fig. 1E to H).

10.1128/mBio.01304-21.3 FIG S3 Capsule production in inactivated *psrA* mutants. (A and B) Amounts of (A) whole-cell and (B) cell wall CPS using the uronic acid assay. Download FIG S3, TIF file, 0.09 MB. Copyright © 2021 Zhang et al. 2021 Zhang et al. https://creativecommons.org/licenses/by/4.0/ This content is distributed under the terms of the Creative Commons Attribution 4.0 International license.

### The inversion of inverted repeats catalyzed by PsrA is influenced by deletion of *fabT*.

**FIG 5:**
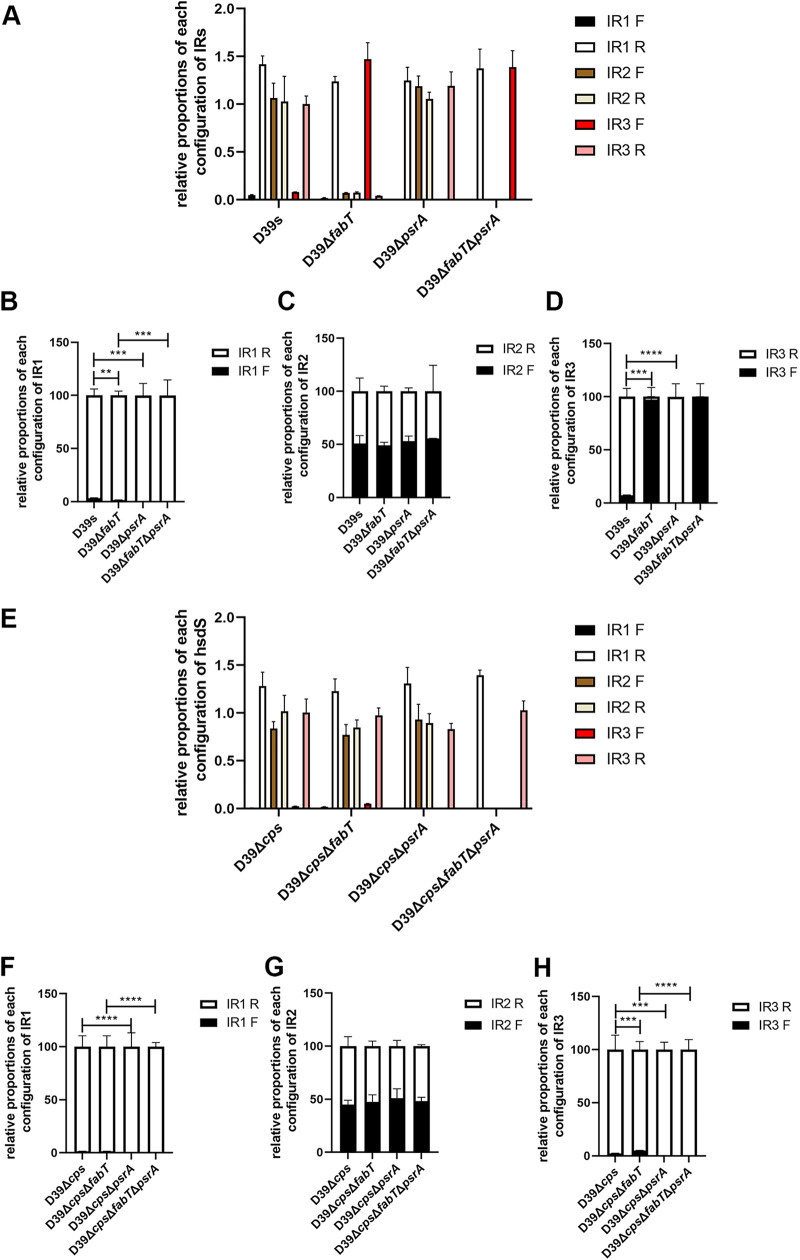
Impact of PsrA and FabT on the inversion forms of IR1, IR2, and IR3. (A to H) Configurations (forward and reverse) of the target sequence flanked by (B and F) IR1, (C and G) IR2 (D and H), and IR3 in (A to D) encapsulated and (E to H) unencapsulated strains were determined by qPCR using 20 ng genomic DNA as the templates. Relative abundance of the configuration of each IR was normalized to IR3R in (A) D39s or (E) D39Δ*cps*, respectively. The sum of 2ΔΔ*C_T_*_-For_ and 2ΔΔ*C_T_*_-Rev_ of each pair of inverted repeats in panels A and E was defined as 100%, and relative compositions of each orientation were compared in (B to D) encapsulated and (F to H) unencapsulated strains. Data are shown as the mean ± SEM of a representative experiment. Each experiment was replicated at least three times. **, *P* < 0.01; ***, *P* < 0.001; ****, *P* < 0.0005.

A recent study has shown that inactivation of recombinase PsrA resulted in the loss of all IR3-mediated inversion events and a partial loss of IR2- and IR1-mediated inversions (19). We therefore examined these inversion events in our experimental strains (Fig. S4A). We found that both encapsulated and unencapsulated Δ*psrA* mutants lacked significant levels of the inverted IR1-bound sequences. The encapsulated strains of D39s generated 3.2% of the forward configuration of IR1, while the level for the Δ*fabT* mutant was 1.5% (Fig. 5B). The levels for the Δ*cps*Δ*fabT* mutant were similar to those of D39Δ*cps* (1.5%) (Fig. 5F). These results suggested that IR1-mediated inversion was not related to the upregulated expression of *psrA* in the Δ*fabT* mutant but also was consistent with the finding that PsrA is indispensable for inversions of sequences bound by IR1 (22, 23).

10.1128/mBio.01304-21.4 FIG S4 Detection of IRs mediated inversion. (A) Positions of the primers used for detection of the orientation of IRs in the SpnD39III locus. (B) Primers for detecting IR inversion and corresponding *hsdS* alleles. (C and E) The relative abundance of the configuration of each IR was normalized to the IR3R in (C) D39s and (E) D39Δ*cps*. (D and F) Forward and reverse configurations of the target sequence flanked by IR1, IR2, and IR3 in (D) encapsulated and (F) unencapsulated strains. Download FIG S4, TIF file, 0.3 MB. Copyright © 2021 Zhang et al. 2021 Zhang et al. https://creativecommons.org/licenses/by/4.0/ This content is distributed under the terms of the Creative Commons Attribution 4.0 International license.

The inversion of IR3 was also completely abrogated in both encapsulated and unencapsulated Δ*psrA* mutants. This suggested that PsrA completely catalyzed the IR3-mediated inversions (Fig. 5D and H). In encapsulated strains, the form of IR3-mediated inversion in the reverse orientation (92.6%) in D39s switched to forward (97.3%) in the Δ*fabT* mutant. The reverse configuration of IR3-mediated inversion was also fully abrogated in Δ*fabT*Δ*psrA* (Fig. 5D). In unencapsulated strains, the forward configuration of IR3 was also increased in the *fabT* strain from 2.2 to 4.9% (Fig. 5H). Likewise, IR3-mediated inversions were not present in the Δ*psrA* mutant. Moreover, the inversion of IR3-bound sequences was partially restored in the complemented strain, indicating that the elevated levels of PsrA that were regulated by FabT were responsible for the IR3-mediated inversion (Fig. S4D).

**FIG 6:**
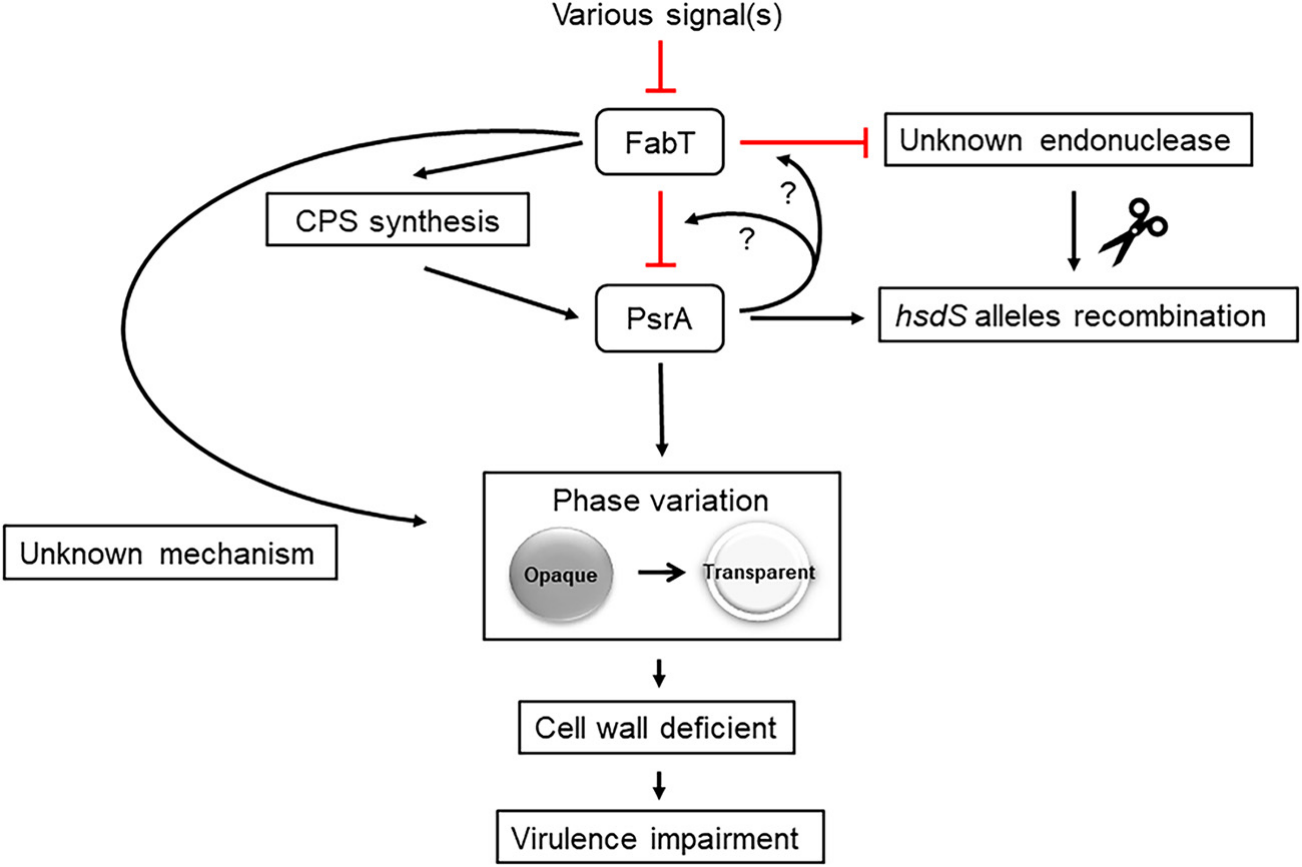
A model of repressor FabT and its modulation of pneumococcal capsule production and colony phase. Once the expression of FabT is repressed by unspecified signals, the production of CPS of S. pneumoniae is significantly downregulated. Meanwhile, FabT is involved in the negative feedback regulation of PsrA and an unknown endonuclease by unknown mechanism. Overexpressed PsrA acts to catalyze the recombination of *hsdS* alleles as well as the phase variation of colony morphology, which seems to be irrelevant with the specific *hsdS* configuration. Besides the PsrA-mediated phase variation, downregulated FabT is able to drive the opaque colony phase to transparent phase directly by unknown mechanism.

In contrast, mutation of *psrA* did not affect the inverse configuration for IR2. The ratio of IR2F to IR2R was 50.8 to 49.1% in D39s and 53.0 to 47.0% in the *psrA* mutants (Fig. 5C and G). Additionally, levels of IR2 fragments were significantly decreased for the encapsulated FabT mutants regardless of fragment direction. Moreover, the IR2 fragments could not be amplified in the D39Δ*fabT*Δ*psrA* mutant (Fig. 5A). This result was confirmed by replacing primer Pr1974 with Pr2042 (data not shown). Moreover, we determined that the IR2.1 downstream sequence was recombined into the genome but not excised due to the existence of a TRD2.1 fragment using primers pr2013/1975 (Fig. S5). However, the matter was more complex because in the unencapsulated strain, the content and the proportions of IR2-mediated inversion were consistent with the parental strain for the Δ*cps*Δ*fabT* mutants, while the IR2 fragment disappeared in strains Δ*cps*Δ*fabT*Δ*psrA*, consistent with D39Δ*fabT*Δ*psrA* (Fig. 5E). This was most likely the result of the action of an unknown endonuclease or recombinase in TRD2.1 ectopic recombination. The observed disappearance of the IR2 fragment also paralleled PsrA mRNA levels. Thus, there may be an unknown endonuclease/recombinase which was regulated by both FabT and PsrA in the same way as PsrA. When the endonuclease was highly expressed, the sequence downstream of IR2.1 was sheared and ectopic integration was favored (Fig. 6).

10.1128/mBio.01304-21.5 FIG S5 Steady-state mRNA levels of TRD2.1 quantified using qRT-PCR. (A) Schematic map representing the *hsdSA* variant with primers indicated. (B) qRT-PCR detection of TRD2.1. Download FIG S5, TIF file, 0.09 MB. Copyright © 2021 Zhang et al. 2021 Zhang et al. https://creativecommons.org/licenses/by/4.0/ This content is distributed under the terms of the Creative Commons Attribution 4.0 International license.

### The site-specific recombinant of the SpnD39III locus is altered in *fabT*-deficient mutants.

**FIG 7:**
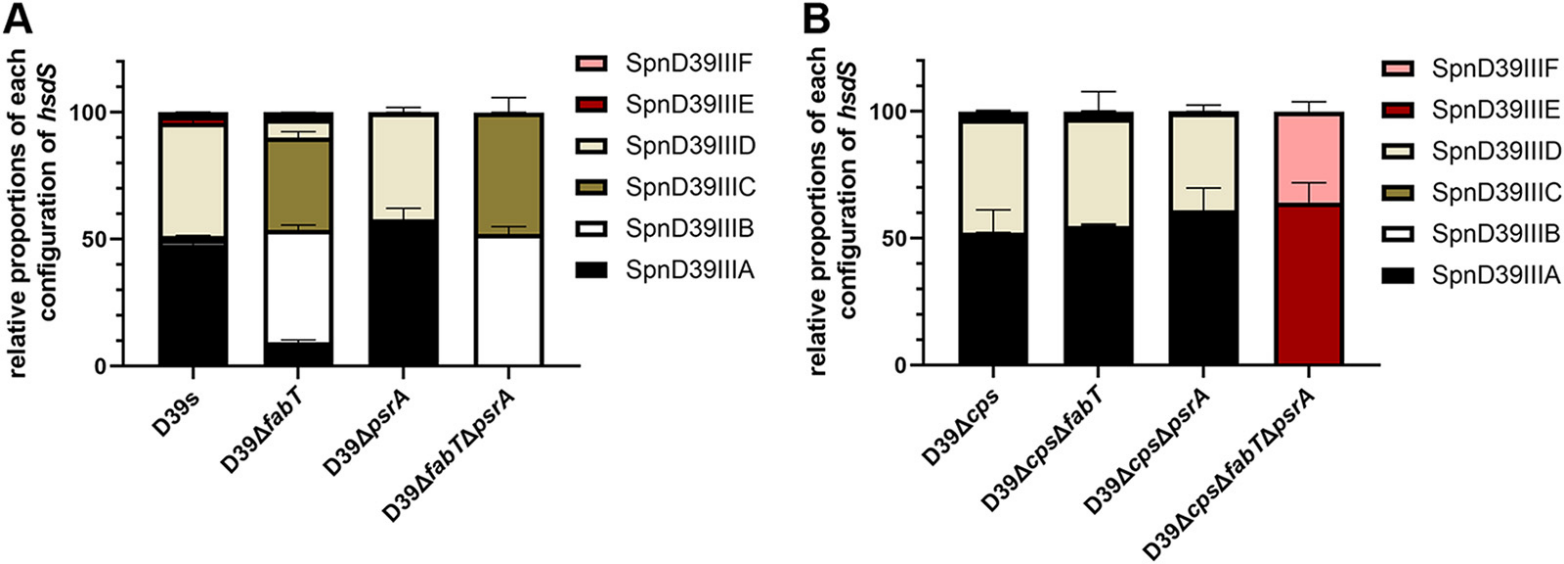
Impact of PsrA and FabT on the *hsdS* allelic configuration. (A and B) Relative proportions of *hsdS* allelic genes in (A) encapsulated and (B) unencapsulated D39 derivatives were determined by qRT-PCR with cDNA as the templates. The transcripts of the 5′ noninvertible sequence of *hsdS* were detected in all strains as a reference to calculate the Δ*C_T_* values. Δ*C_T_* values of each *hsdS* allele were normalized again with the mean value of *hsdS*A as a reference in each strain to obtain ΔΔ*C_T_*. The sum of the ΔΔ*C_T_* of all six *hsdS* alleles was defined as 100%, and the relative composition of each *hsdS* allelic gene was calculated. Primers are listed in Fig. S6.

Recombinase PsrA catalyzes the inversion of IRs and mediates recombination of the 3 homologous *hsdS* genes. The recombination process results from the rearrangement of 5 different TRDs within homologous *hsdS* genes and was associated with distinct DNA methylation patterns. Recent studies revealed the relationship between *hsdS* allelic inversion and methylation of distinct DNA motifs in genomes catalyzed by the type I RM system SpnD39III. The different methylomes led to the reversible switching between O and T colony phases, although previous results were not consistent with each other (19–21). To evaluate the existence of shuffled TRDs, we designed a qRT-PCR assay that amplified distinct sequences on the mRNA of each allelic *hsdS* gene to evaluate the frequency of *hsdS* inversions in the SpnD39III locus using a 5′ noninvertible region shared by the six *hsdS* alleles as an internal reference (Fig. S6). We confirmed that the expression levels of *hsdM* that lies upstream of *hsdS* and is cotranscribed with *hsdS*, was not affected in *fabT* and *psrA* mutants (Fig. S2C). These results demonstrated that the D39s stocks held within our laboratory collection expressed a mixture of the SpnD39III variants. SpnD39IIIA (47.9%) and D (44.8%) were the predominant variants in D39s, whereas SpnD39IIIB (44.2%) and C (36.5%) were predominant for the Δ*fabT* mutant. In the absence of the PsrA recombinase, only SpnD39IIIA/D or SpnD39IIIB/C were detected in D39s and Δ*fabT* (Fig. 7A).

10.1128/mBio.01304-21.6 FIG S6 Methods used for hsdS allelic detection. (A) Schematic map of the organization in the SpnD39III locus and six alternative *hsdS* genes. (B) The proportions of *hsdS* alleles in the mixed population. The red line indicates the two transcripts in the SpnD39III locus. The cDNA of the mixed population was used as the template. (C) Primer pairs used to detect the *hsdS* alleles. Download FIG S6, TIF file, 0.2 MB. Copyright © 2021 Zhang et al. 2021 Zhang et al. https://creativecommons.org/licenses/by/4.0/ This content is distributed under the terms of the Creative Commons Attribution 4.0 International license.

In unencapsulated stains, the levels for D39Δ*cps* were consistent with the presence of *hsdS* alleles in D39s in which SpnD39IIIA (50.9%) and D (44.6%) were the predominant variants. The Δ*cps*Δ*fabT* double mutant displayed no deviation with SpnD39IIIA (52.8%) and D (42.4%). However, in the Δ*cps*Δ*fabT*Δ*psrA* mutant, the formation of the *hsdS* allele was locked in SpnD39IIIE (64.0%) and F (35.9%). In the different *psrA* mutants, the *hsdS* gene was locked in all six *hsdS* alleles, indicating that the intrastrain phase variation was dependent on the activity of invertase PsrA (Fig. 7B).

### The Δ*fabT* mutant impaired pneumococcal virulence.

**FIG 8:**
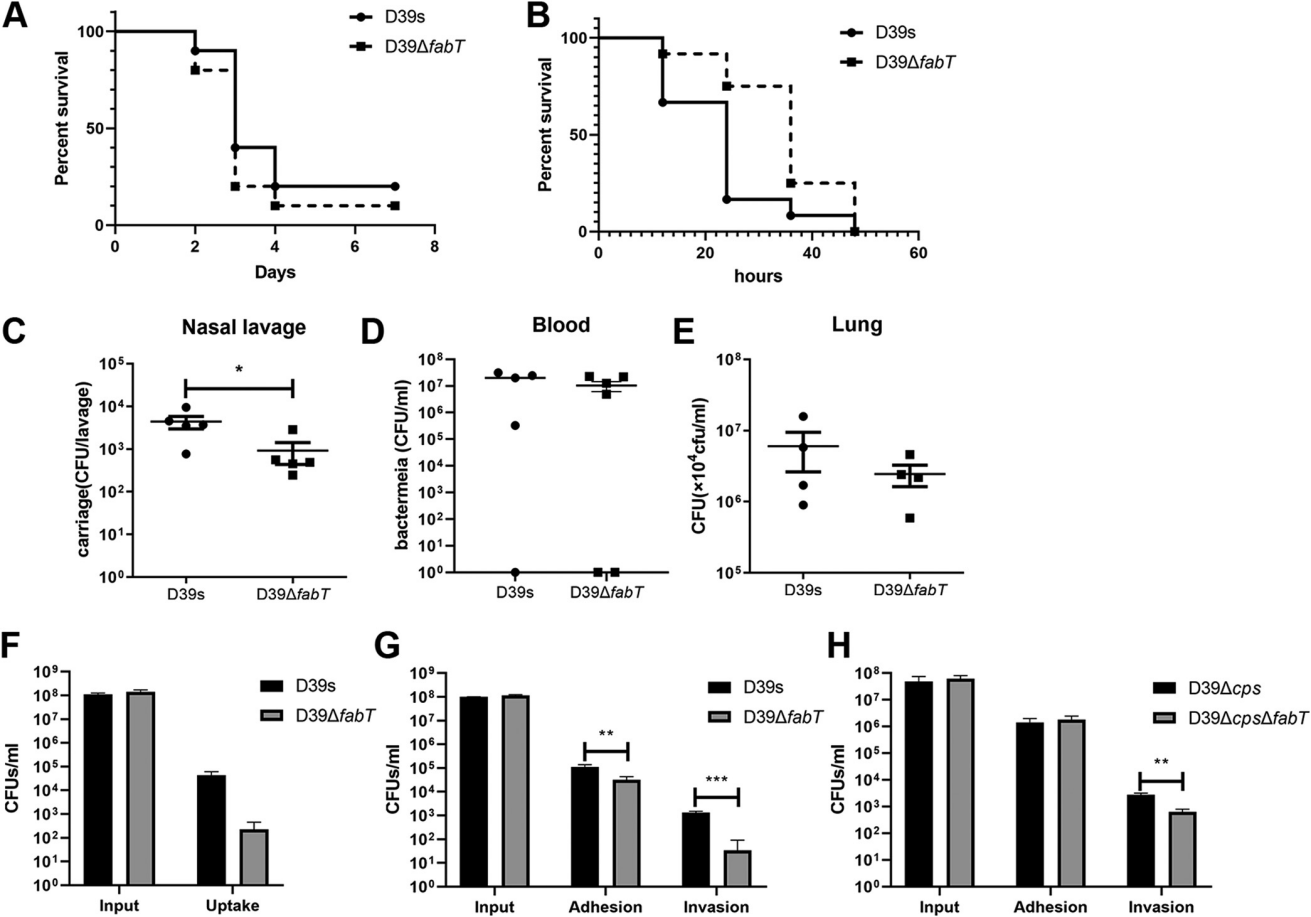
*In vivo* and *in vitro* phenotypes of *fabT* mutant strains. (A) Survival experiments were performed by intranasal inoculation of 1 × 10^8^ CFU S. pneumoniae into C57BL/6 mice. (B) Survival in the mouse septicemia model. Mice were injected intraperitoneally with 776 and 600 CFU of strains D39s and D39Δ*fabT*, respectively. (C to E) Intranasal infection with 1.5 × 10^7^ CFU of S. pneumoniae. Bacterial loads were evaluated by cultures of (C) nasal lavage, (D) blood, and (E) lung homogenates. (F) Infection of mouse peritoneal primary macrophages with D39s and D39Δ*fabT*. MOI = 100:1. The input and the intracellular (uptake) bacteria were counted by plating from serial dilutions. (G and H) A549 epithelial cells were infected at an MOI of 100:1 with (G) D39s and (H) D39Δ*cps*. Input, cell-associated (adhesion), and intracellular (invasion) bacteria were counted by plating from serial dilutions.

Recent studies demonstrated that distinct SpnD39III variants diversify the capacity of pneumococcal adhesion and invasion in the upper respiratory tract and for nasopharyngeal colonization (19–21). We used a mouse intranasal infection assay to investigate the role of FabT in S. pneumoniae D39. Mice that were intranasally infected with Δ*fabT* showed an improved survival rate compared to the parental S. pneumoniae D39s strain, while mice infected with D39s appeared visibly sick and had considerable weight loss, yet the difference was not statistically significant (Fig. 8A). During invasive infection, the numbers of bacteria in the nasal lavage fluid from Δ*fabT*-infected mice were lower than those in the WT-infected mice at 48 h after challenge (Fig. 8C). However, the bacterial loads in their blood or lung homogenates did not show significant differences (Fig. 8D and E). A slightly lower virulence was also present in the bacteremia model (Fig. 8B). The most important pathogenic determinant of S. pneumoniae is the thick pneumococcal capsule that minimizes phagocytosis. We also evaluated the bacterial survival rate after incubation with human macrophages in the absence of serum. To our surprise, the Δ*fabT* mutant showed an increase in antiphagocytosis against macrophages (Fig. 8F). The adherence of the Δ*fabT* mutant to the epithelial A549 cells was significantly decreased compared to D39 (Fig. 8G). This was consistent with the decrease in bacterial loads in nasal lavage specimens of the Δ*fabT* mutant. To avoid potential impact of the capsule, unencapsulated strains were used to verify the adhesion capacity. The unencapsulated Δ*fabT* mutant displayed a lower level of invasion (Fig. 8H). Taken together, these results indicated that FabT attenuated the virulence of S. pneumoniae by altering the phenotypic variation, and this had a significant impact on pneumococcal colonization and persistence.

## DISCUSSION

This work demonstrated that mutation of the transcriptional repressor FabT in the S. pneumoniae D39 strain led to an unexpected decrease in capsule production that was accompanied by a novel rough colony morphology on blood agar plates. Complementation of the *fabT* strain restored capsule production and normal colony morphology. Although the capsule production for the D39Δ*fabT* mutant was significantly impaired, the expression levels from the *cps* locus were unaffected. It is unknown how the fatty acid biosynthesis transcriptional regulator FabT impacts the pneumococcal capsule. A site-specific recombinase PsrA was identified in transcriptome analysis, and this played a dominant role in specific alleles of the SpnD39III (Spn5556II) type I RM system and contributed to TRD rearrangements of *hsdS* alleles that further affected colony phase variation. Capsule production is an enormous energetic burden for the cell and can compete directly with central metabolism for energy (30, 38). The encapsulated Δ*fabT* mutant might be under higher metabolic stress when its CPS and fatty acid production are altered. Thus, in the absence of an energy demand by CPS production, the demand of altered expression of PsrA to regulate the SpnD39III epigenetic switching machine was much less for the unencapsulated Δ*fabT* mutant (Fig. 6). Our initial expectation was that the differences in the amount of CPS correlated with the colony morphology in the *fabT*-deficient strain, and this has been demonstrated previously (6, 39). However, we found that 100% O *psrA* mutants produced CPS at the same level as the parental strain. This confirmed that CPS production was not only affected by the variation of colony opacity, but FabT deletion was able to reduce the capsule thickness through an unknown mechanism (Fig. 6). This result was supported by previous experiments what utilized fixed SpnD39III alleles that were transformed into the hyper-encapsulated Δ*adcAII* mutant and were unable to alter that phenotype (32).

Our results demonstrated that the highly expressed recombinase PsrA contributed to the presence of more T colonies in the Δ*fabT* mutant. In agreement with previous studies, the *psrA* frameshift-deficient mutant strains diminished the tendency to generate T colonies and were fully restored to the O colony phase in both encapsulated and unencapsulated parental strains. Therefore, deletion of FabT may promote the T colony phase of S. pneumoniae by transcriptional activation of recombinase PsrA (Fig. 6).

To further investigate the underlying mechanism, we examined IR inversions and the resulting disposition of *hsdS* alleles catalyzed by PsrA at the SpnD39III locus. We found that the absence of PsrA completely abrogated the inversion of IR1- and IR3-mediated TRDs, and the inversion mediated by IR2 was not affected. This outcome conflicted with previous reports that had indicated that the absence of PsrA in the D39 derivative eliminated all IR3 shuffling and partially affected the inversion of IR2 while leaving inversions mediated by IR1 intact (22). A complete dependence of IR3 on PsrA as well as IR1- and IR2-mediated inversions has also been reported (23).

As the recombination within the S. pneumoniae SpnD39III loci leads to epigenetic regulation of genome DNA methylation, we further investigated the composition of the mRNA of *hsdS* alleles. However, our results were not consistent with the IR inversions detected using genomic DNA (gDNA). All TRD recombinations that shuffled between IR3 were halted in *psrA* frameshift mutants (i.e., D39Δ*fabT*Δ*psrA* consisted of *hsdSB* and C variants, and IR3 was fully in the forward configuration). The configuration of IR1 conflicts with our IR detection results and *hsdS* quantification results (i.e., D39s contained approximately 50% *hsdSA* variants, which were IR1 forward configuration). One potential explanation was that the transcription occurred at the *hsdS*″ gene that led to mRNA amplicons that were not solely present in the transcript of a specific *hsdS* variant. The transcription of *hsdS*″ has been previously verified (23).

Another potential explanation was the ectopic recombination generated from direct repeats (DRs) mediated excision on the *hsdS* genes. Li et al. found that besides three pairs of IRs mediating *hasS* recombination events, two pairs of DRs can mediate *hsdS* deletion events (19). Likewise, we found that the detection fragments for IR2 that included DR2 were lost in FabT and PsrA double knockouts. However, we do not as yet understand the reason for the loss of IR2 in the Δ*fabT* and Δ*fabT*Δ*psrA* mutants. Recently, the presence of an uncharacterized recombination system in the S. pneumoniae genome was hypothesized to play a role in PsrA-independent inversions (22, 23). Our results strongly imply that there are unknown mechanisms or specific recombination systems playing an important role in SpnD39III locus inversion. Δ*fabT*, Δ*fabT*Δ*psrA*, and Δ*cps*Δ*fabT*Δ*psrA* mutants displayed significant increases in *psrA* expression levels, and the decreased IR2 fragment levels indicated a similar trend. Thus, we hypothesized that this unknown recombinase can mediate the ectopic integration of the sequence downstream of IR2.1 into other homologous positions on the genome in the absence of *fabT* and that FabT may work as a repressor of the unknown recombinase in the same manner as PsrA (Fig. 6). The precise mechanism or a specific recombination system of the FabT-dependent recombination awaits further investigation.

The relationship between the locked *hsdS* allele and the colony opacity were quite different in recent studies. For instance, Li et al. found that only *hsdSE* allele strains were opaque and the others led to transparent variants (19). Manso et al. found that the *hsdSA*, *E* allele strains were opaque (20), and not any of them were completely transparent. Oliver et al. demonstrated that *hsdSA* and *B* alleles were completely opaque, while the others were transparent (21). Our results of quantitatively detected *hsdS* alleles demonstrated that D39Δ*psrA* and Δ*cps*Δ*psrA* mutants were locked in *hsdSA* and *D* alleles; the Δ*fabT*Δ*psrA* mutant was locked in *hsdSB* and *C* alleles, whereas the Δ*cps*Δ*fabT*Δ*psrA* mutant was locked in *hsdSE* and *F* alleles. The presumed mechanism of phase variation regulation by the SpnD39III system might be affected directly by recombinase PsrA rather than the unique sequence specificity protein HsdS that mediated variations in genome DNA methylation patterns, though this needs further investigation.

TRD shuffling at the SpnD39III locus was also controlled by a PsrA-independent mechanism, though recent studies have ruled out site-specific or RecA-mediated recombination in S. pneumoniae (22, 23). The gene encoding a DNA mismatch repair protein, MutS, was slightly upregulated in our D39Δ*fabT* mutant, indicating a putative mechanism for inversion repair. However, the qRT-PCR results did not support this hypothesis (Fig. S2D).

Existing evidence strongly suggests that PsrA is indirectly regulated by FabT. FabT is a MarR family transcription factor, and the latter are characterized by possession of a conserved winged-helix-turn-helix (wHTH) DNA-binding motif that is widespread in all prokaryotes from archaea to bacteria (40). FabT globally regulates the fatty acid synthesis pathway via binding the distinct DNA palindromes (ANTTTGACTGTNAAATT) located in the promoters upstream of FAS genes (24, 27, 41). Our study demonstrated that the expression level of *psrA* was significantly upregulated in the *fabT* deletion mutant, but there was no consensus on predicted DNA palindrome sequences located in the promoter region of *psrA.*

FabT is a transcriptional regulator in the *fab* gene cluster and thus regulates the transcription of fatty acid synthesis and controls the membrane fatty acid composition in S. pneumoniae (24, 42, 43). The role of FabT in the regulation of fatty acid biosynthesis is well established. Opaque and transparent variants carry the same types of fatty acids but differ in their proportions (28). The opaque variants possess a relatively lower degree of UFAs in the cell membrane. The *fabT* knockout can also upregulate the entire *fab* gene cluster, with the exception of *fabM*, and leads to decreased levels of UFAs coupled with an increase of saturated fatty acids (SFAs) (24). However, there is a putative FabT-binding sequence upstream the *fabM*, and FabT can generate a FabT-DNA complex with the FabT-binding site upstream of *fabM* (31). This indicated that FabT can regulate *fabM* expression. Indeed, we found that *fabM* expression levels were increased using both transcriptome and qRT-PCR analyses. FabM is an essential enzyme responsible for UFA synthesis in pneumococci. Therefore, a possible explanation for the higher proportion of transparent variants might be associated with the upregulated *fabM* in the D39Δ*fabT* mutant.

Colonization of the nasopharyngeal mucosa is an essential initiative step in the pathogenesis of pneumococcal disease and involves direct interaction between pneumococcal adhesins and specific receptors on host epithelial cells. During colonization, S. pneumoniae expresses low levels of CPS (transparent type) to enhance the exposure of cell surface proteins and promote binding to epithelial cells. In contrast, S. pneumoniae expresses high-level CPS (opaque type) during systemic infection to evade complement-mediated opsonophagocytosis (6). Based on the colony morphology and impaired capsule phenotypes we observed in the *ΔfabT* mutant, we hypothesized that deletion of *fabT* most likely resulted in enhanced adherence to epithelial cells and increased nasopharynx colonization in mice. However, the result was quite counterintuitive since the encapsulated Δ*fabT* mutant displayed reduced adherence to A549 cells and colonization in the mouse nasopharynx. These results suggested that in addition to the reduction of the capsule caused by the phase variable Δ*fabT* mutant, other components of surface structures related to adhesion and invasion, such as teichoic acids or surface proteins, were also affected.

Although the precise intermediate steps remain to be identified, the results of the present study describe a new function of transcriptional regulator FabT during the process of phase variation and the expression of capsule, resulting in attenuated bacterial virulence. The site-specific recombinase PsrA in the type I RM system SpnD39III was able to catalyze the inversion of IR1 and IR3 but not IR2. The phenotypic variation depends only on the activity of PsrA, not the recombination of *hsdS* alleles. FabT is essential for the negative feedback regulation of PsrA and an unknown endonuclease. The underlying mechanism of how FabT affects the recombinase PsrA and what virulence factors besides capsule are affected in the different variation phases will be further investigated in our future study.

## MATERIALS AND METHODS

### Strains and growth conditions.

The strains used in this study are derivatives of strain D39 (Table S2). For growth in liquid cultures, S. pneumoniae strains were incubated in Todd-Hewitt broth (Difco, BD Diagnostics, Sparks, MD, USA) or supplemented with 0.5% yeast extract (THY medium) broth at pH 7.4 unless otherwise stated. For transformation, cultures growing in C+Y medium (C medium supplemented with 0.8% yeast extract) (pH 8.0) were induced with synthetic competence-stimulating peptide (CSP-1; China Peptides, Shanghai, People’s Republic of China) as previously described (44). For growth on solid medium, blood agar plates (Chongqing Pangtong, Chongqing, People’s Republic of China) or tryptic soy agar (TSA; BD Diagnostics) were used. Broth and plates were cultivated at 37°C with 5% CO_2_. Antibiotic selection was used at the following concentrations: streptomycin, 150 μg/ml; kanamycin, 200 μg/ml; spectinomycin, 50 μg/ml for Escherichia coli and 200 μg/ml for S. pneumoniae.

10.1128/mBio.01304-21.8 TABLE S2 Bacterial strains and plasmids used in this study. Download Table S2, PDF file, 0.1 MB. Copyright © 2021 Zhang et al. 2021 Zhang et al. https://creativecommons.org/licenses/by/4.0/ This content is distributed under the terms of the Creative Commons Attribution 4.0 International license.

### Strain constructions.

The construction of pneumococcal mutants was carried out in D39s, a streptomycin-resistant derivative of strain D39 (45). Unencapsulated D39Δ*cps* mutants were constructed by unmarked deletion of the entire *cps2A* promoter region *dexB-cps2A* as previously described (46). In brief, the up- and downstream sequences of the *dexB-cps2A* intergenic region were amplified with primer pairs Pr1901/1902 and Pr1903/1904 from D39s, and the new Janus cassette (referred to as JC1) was amplified with Pr1905/1906 from the strain TH7457 (19). The overlap extension fusion PCR was performed with Pr1901/1904 before being transformed into D39s to generate D39Δ*dexB-cps2A*::*JC1*. The up- and downstream sequences of the *dexB-cps2A* region were then amplified with Pr1901/1907 and Pr1908/1904 from D39s and fused by overlap primer Pr1901/1904. The fusion PCR product was transformed into D39Δ*dexB-cps2A*::*JC1* to generate unmarked D39Δ*cps* mutant.

To construct the unmarked deletion mutant Δ*fabT*, first the upstream (primers Pr1917/1910) and downstream (primers Pr1913/1914) sequences of the *fabT* gene were fused with JC1 (primers Pr1911/1912) with primers Pr1917/1914 and transformed into the background strain to generate the Δ*fabT*::*JC1* mutant. Strain Δ*fabT* was then constructed by transforming the amplicon of the fusion PCR product, in which upstream (primers Pr1917/1915) and downstream (primers Pr1916/1914) sequences were overlapped with primers Pr1917/1914.

The complemented FabT was constructed by using suicide vector pPEPZ (47), which integrates efficiently in S. pneumoniae. Through the homologous fragment carried on the plasmid, the insert can be integrated upstream of spd_1736 and is a nonessential pseudogene. Upstream of the pPEPZ multiple cloning site, the expression of the insert is regulated by a P_lac_ promoter, and the lack of LacI repressor in S. pneumoniae enabled constitutive expression from the construct. The sequence *fabT*-FLAG3 was amplified with primers Pr2043/2034 and Pr2035/2044 and then overlapped with Pr2043/2044. The plasmid and DNA fragment were digested with BglII and XhoI, followed by ligation with T4 DNA ligase. To construct the complementary strains, the resulting plasmid pPEPZ-P_lac_-RBS-*fabT*-FLAG3 was used to transform into the Δ*fabT* mutants.

For the *psrA* frameshift mutants, in order to create the mutant in all six *hsdS* alleles, the homologous sequence was amplified in the intergenic region of IR3.1-IR3.2 (primers Pr1994/2010 and Pr2013/1975) that does not undergo inversion. After fusion PCR of JC1 (primers Pr2011/2012) with primers Pr1994/1975, the amplicon was transformed into the background strains to insert the JC1 into the invertase PsrA. The frameshift mutant sequence was amplified with Pr1994/2008 and Pr2007/1975. The resulting fusion PCR amplicons were transformed into the corresponding JC1 replacement derivatives to generate *psrA* frameshift mutants (22). All of the primers used in this study are listed in Table S3. The transformation of S. pneumoniae was carried out by natural transformation essentially as previously described (48).

10.1128/mBio.01304-21.9 TABLE S3 Primers used in this work. Download Table S3, PDF file, 0.04 MB. Copyright © 2021 Zhang et al. 2021 Zhang et al. https://creativecommons.org/licenses/by/4.0/ This content is distributed under the terms of the Creative Commons Attribution 4.0 International license.

### Fluorescein isothiocyanate (FITC)-dextran exclusion assay.

The thickness of capsule was determined by measuring the zone of exclusion of FITC-dextran (2,000 kDa; Sigma, St. Louis, MO, USA) based on published methods (49, 50). In brief, bacteria were cultured in 5 ml THY, harvested during the exponential growth phase by centrifugation at 3,000 × *g* for 5 min, and washed once with phosphate-buffered saline (PBS), and 20 μl of bacteria was mixed with 2 μl of a 10-mg/ml stock solution of FITC-dextran for staining. The stained sample was mounted on slides and visualized using a ×100 objective.

### Immunofluorescence microscopy.

Capsular polysaccharides of the D39s and D39Δ*fabT* mutant were detected by immunofluorescence microscopy as previously described (51). Briefly, pneumococcus type 2 serum (SSI Diagnostica, Denmark) was used as the primary antibody at a dilution of 1:50, and goat anti-mouse IgG-PE (Santa Cruz Biotechnology, Santa Cruz, CA, USA) was used as the secondary antibody at a dilution of 1:500. Finally, images were obtained with a Nikon Eclipse 80i microscope.

### Transmission electron microscopy (TEM).

To determine the capsule morphology, bacteria were grown to an optical density at 600 nm (OD_600_) of 0.5 in THY and harvested by centrifugation. The bacterial pellets were fixed with 2.5% glutaraldehyde for 24 h, embedded into 2% agarose, and processed by the Electron Microscopy Research Service of Chongqing Medical University. Capsule thickness was determined by measuring 20 randomly chosen cells using Image J software (https://imagej.nih.gov/ij/download.html).

### Capsule preparation and quantification of the glucuronic acid content.

Samples for the determination of the capsular glucuronic acid amounts were prepared as follows. Bacteria were grown in THY under semiaerobic conditions as described above. The whole-cell CPS were prepared in PBS, and cultures were harvested at an OD_600_ of 0.5. An aliquot of 6 ml was pelleted at 6000 × *g* for 5 min and washed with PBS and then suspended in 500 μl Tris-HCl, pH 7, and 1 mM MgSO_4_ as whole-cell CPS samples. To determine the amount of CPS attached to the cell wall, pellets were suspended in 1 ml PBS with 2% wt/vol SDS preheated to 100°C. Cells were heated at 100°C for 30 min. The cell walls were washed 3 times in PBS to completely remove SDS and then suspended in 300 μl Tris-HCl/MgSO_4_ buffer, treated with 40 units of mutanolysin (Sigma), and incubated overnight at 37°C. The samples were then treated with 100 μg proteinase K at 56°C for 4 h. The glucuronic acid of the cell wall-associated CPS or whole-cell CPS was quantified using the method for quantitative determination of uronic acids as previously described (52).

### RNAseq analysis.

The RNAprotect bacterial reagent and RNeasy Protect bacterial kit (Qiagen, Hilden, Germany) were employed for RNA extraction according to the manufacturer’s instructions. RNAseq was performed at Novogene Bioinformatics Technology (Beijing, People’s Republic of China). Trimmed reads were mapped to the genome of S. pneumoniae D39 as the reference genome (GenBank Accession number NC_008533.2). Differential expression analysis of two groups (two biological replicates per group) was performed using the DESeq R package 1.18.0. The resulting *P* values were adjusted using Benjamini and Hochberg’s approach for controlling the false-discovery rate. Genes with an adjusted *P* value of <0.05 found by DESeq were assigned as differentially expressed.

### qRT-PCR.

RNA extraction was performed as described above. PrimeScript RT master mix (TaKaRa, Beijing) was used to prepare cDNA from total RNA according to the manufacturer’s instructions. The primers used in this study are listed in Table S3. *gyrB* was amplified with primers Pr1930/1931 as an internal control for the constitutively expressed gene. The relative expression level was calculated using the average mean cycle threshold value for the target gene for each sample and *gyrB*. The results of representative experiments are presented as the means of three replicates ± standard deviations.

### Colony phase variation observation.

Observation of pneumococcal colony opacity was carried out with TSA plates supplemented with catalase (Sigma) as described previously (53). Briefly, S. pneumoniae was grown to an OD_600_ of 0.5 in THY. The culture was then diluted with PBS at 1:10,000 to approximately 10^4^ CFU/ml. Then, 50 μl of diluted bacteria was mixed with 100 μl of catalase work solution (20 mg/ml) and spread on TSA plates and incubated in a 5% CO_2_ atmosphere at 37°C for the indicated times. The observation of pneumococcal colonies was carried out with a Nikon Eclipse Ti inverted microscope. The plate was put on the stage with the open side up and an LED spotlight placed diagonally above was used to provide an oblique and transmitted light to observe the colony. Each experiment was repeated at least three times.

### Determination of the inversion configuration of IRs in genomic DNA.

Forward and reverse configurations of IRs in the *hsdS* locus were detected by qPCR as described previously (23). Briefly, the template genomic DNA (gDNA) of each strain was extracted using a TIANamp bacterial DNA kit (Tiangen, Beijing, People’s Republic of China), and 20 ng of genomic DNA was used as the template for each reaction. The primers used to determine the inversions of three IRs are listed in Table S3. To determine the relative configuration of a particular IR, the average cycle threshold (*C_T_*) of each inverted repeat was first normalized by subtracting the *C_T_* of the internal reference Pr2001/1995 to obtain Δ*C_T_*. Δ*C_T_* values for each IR in the forward and reverse states were normalized again with the mean value of IR3R as a reference in each strain to obtain ΔΔ*C_T_.* The relative amplification level was then calculated according to the equation 2ΔΔ*C_T-_*_For_ and 2ΔΔ*C_T_*_-Rev_, and the total amplification level was defined as 100%, which was the total value of 2ΔΔ*C_T_*_-For_ and 2ΔΔ*C_T_*_-Rev_.

### *hsdS* quantification.

The *hsdS*′ and *hsdS*″ genes lack independent transcription and are almost transcriptionally silent (23). Thus, the specific sequence in the transcript of *hsdS* was quantitated to assess the relative abundance of allelic *hsdS* genes in the mixture population. The cDNA prepared above was used as the template. Quantitative reverse transcription-PCR (qRT-PCR) was performed with primer pairs Pr2005/1974, Pr2005/1970, Pr1992/1970, Pr1994/1974, Pr2005/1971, and Pr1992/1971 to detect the specific sequence in *hsdSA* to -*F*, respectively. As an internal reference for PCR, the 5′ noninvertible region shared by the six *hsdS* alleles was also amplified with primers Pr2001/1995. The amplification efficiency between different primers was almost the same. The average *C_T_* of each *hsdS* allele was first normalized by subtracting the *C_T_* of the internal reference Pr2001/1995 to obtain Δ*C_T_*. Δ*C_T_* values for *hsdS* alleles were normalized again with the mean value of *hsdSA* as a reference in each strain to obtain ΔΔ*C_T_*. The data from one representative experiment are presented as the mean value of triplicate samples ± the standard error of the mean (SEM) for each strain. Each experiment was repeated at least three times.

### Adhesion and antiphagocytic assay.

Pneumococcal adherence and invasion assays with A549 human type II pneumocytes were performed in 24-well plates. Confluent epithelial cells (∼2 × 10^5^ cells/well) were inoculated with 2 × 10^7^ CFU pneumococci at a multiplicity of infection (MOI) of 1:100 and incubated in Dulbecco’s minimal essential medium (DMEM) at 37°C in the presence of 5% CO_2_ for 1 h. Subsequently, the cells were rinsed five times with PBS to remove unbound bacteria. For isolation of pneumococci that were taken up by the cells, extracellular bacteria were killed by treatment with gentamicin (100 μg/ml) and penicillin G (10 μg/ml). The intracellular pneumococci were recovered after lysing of the cells with double-distilled water (ddH_2_O) and plated on blood agar plates with the appropriate dilution. The amount of intracellular surviving bacteria per well was then determined. Each experiment was repeated at least three times. The results of representative experiments are presented as the means of three replicates ± standard errors. Phagocytosis of pneumococci was determined with mouse peritoneal primary macrophages. The remaining steps were the same as described above.

### Mouse infection assays.

Mouse infection assays were performed as previously described (51, 54, 55). Mice were obtained and raised at the experimental animal center of Chongqing Medical University. All the animal experiments were discussed with and approved by the Animal Care and Use Committee of Chongqing Medical University. All procedures were performed according to the recommendations in the Guide for the Care and Use of Laboratory Animals and conformed to animal protection laws of the People’s Republic of China and applicable guidelines. The D39s and D39Δ*fabT* mutant used for infection were grown to an OD_600_ of 0.5 in THY. Bacteria were washed and suspended in PBS. For the lung infection model, C57BL/6 mice (6 to 8 weeks old, male) were infected intratracheally with 1.5 × 10^7^ CFU and 1 × 10^8^ CFU of S. pneumoniae for pneumonia model and survival assay, respectively. To determine the organ involvement, blood aliquots and nasal lavage samples were collected from mice following induction of general euthanasia at 48 h after infection. Lung and spleen whole tissues were homogenized. Bacterial counts in the blood as well as organ homogenates were determined by separately plating serial dilutions. The results were presented as the means of three replicates ± the standard deviation (SD). Mouse survival was monitored daily for 7 days.

For the sepsis model, C57BL/6 mice (6 to 8 weeks old, male) were infected intraperitoneally with 100-μl volumes of S. pneumoniae. The CFU/mouse were 776 and 600 for D39s and the D39Δ*fabT* mutant, respectively. Survival was recorded every 12 h until death.

### Statistical analysis.

All analyses were performed with Prism 8 (GraphPad Software, San Diego, CA, USA). The data were statistically analyzed with two-tailed unpaired Student’s test or the nonparametric Mann-Whitney U test. Statistical significance was defined by *P < *0.05 (*), <0.01 (**), and <0.001 (***).
