# Supplementary figures and images for: Inactivation of Transcriptional Regulator FabT Influences Colony Phase Variation of Streptococcus pneumoniae
Source: mBio. 2021 Aug 17;12(4):e01304-21. doi: 10.1128/mBio.01304-21 (PMC8406281; doi:10.1128/mBio.01304-21)

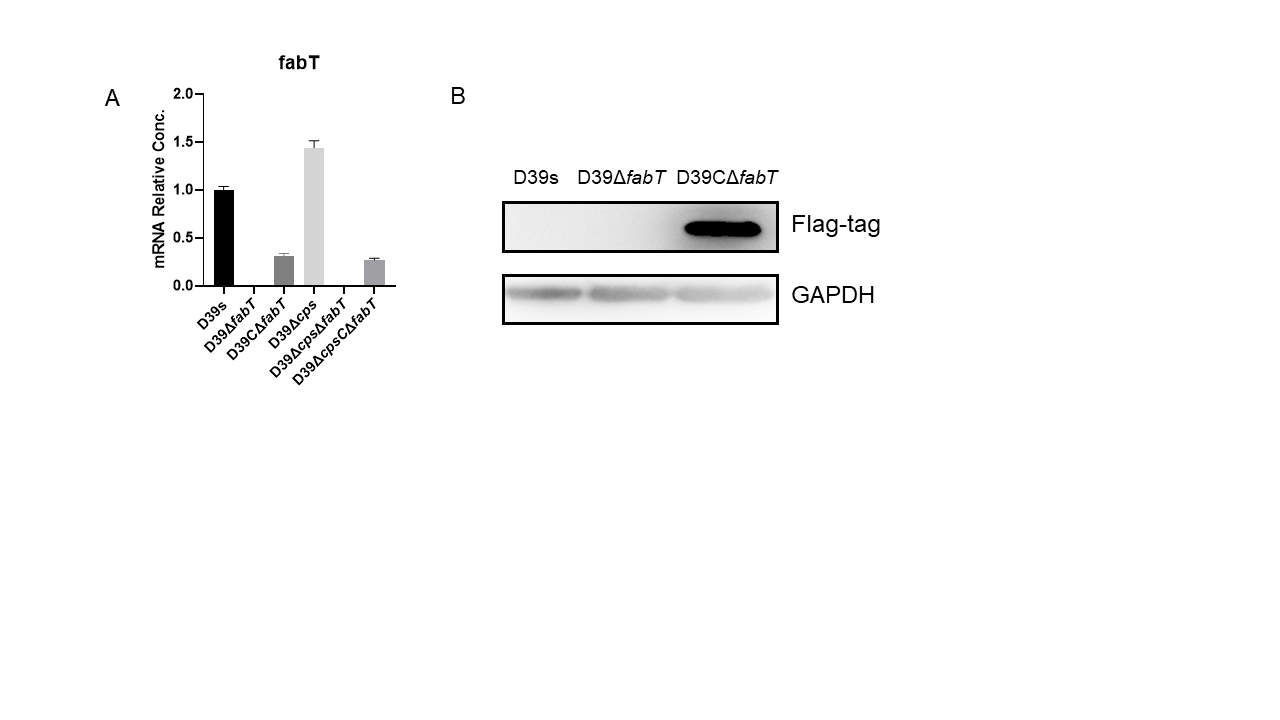

Supplement: FIG S1 [file mbio.01304-21-sf001.tif]

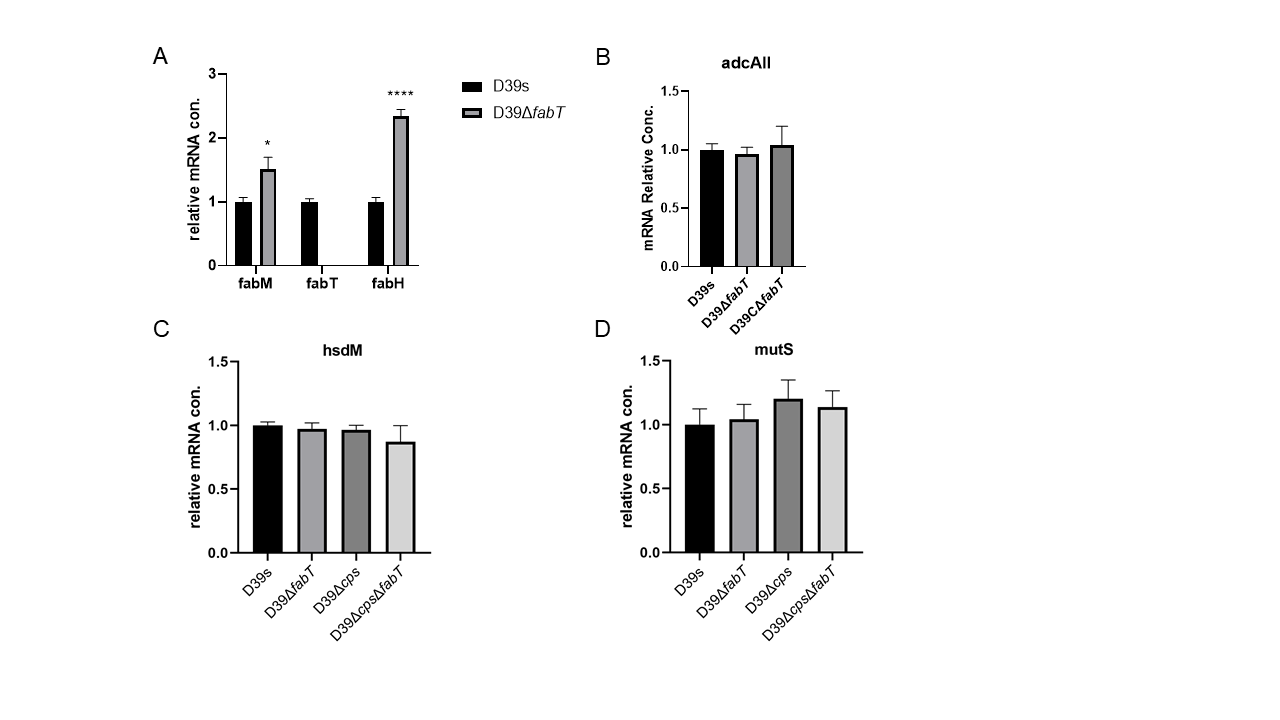

Supplement: FIG S2 [file mbio.01304-21-sf002.tif]

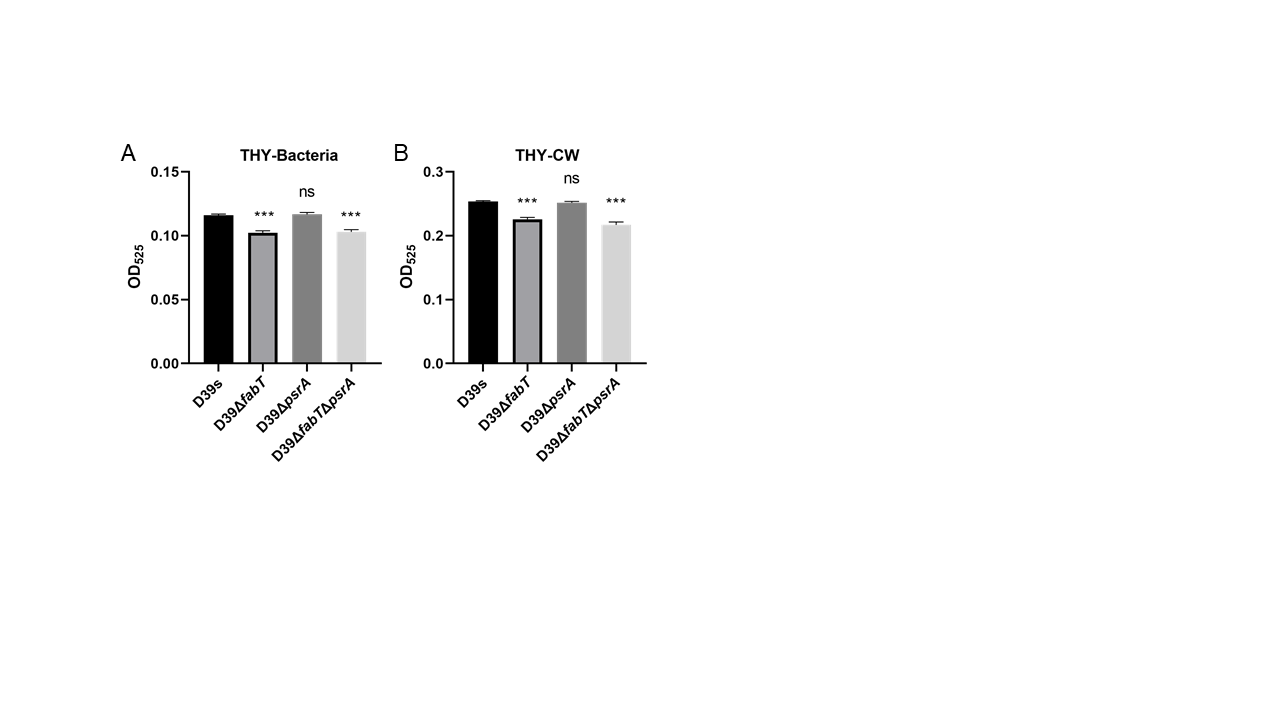

Supplement: FIG S3 [file mbio.01304-21-sf003.tif]

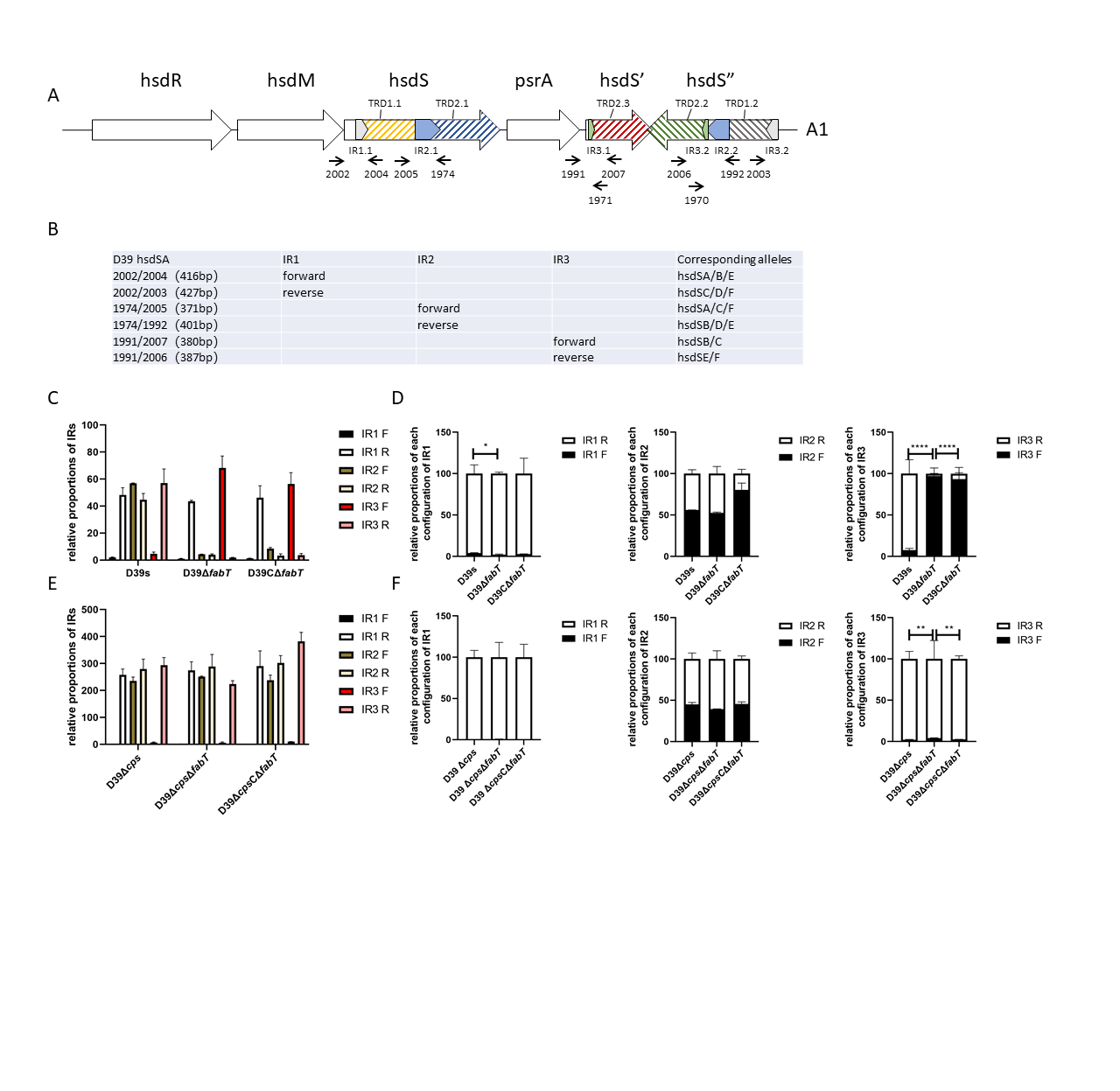

Supplement: FIG S4 [file mbio.01304-21-sf004.tif]

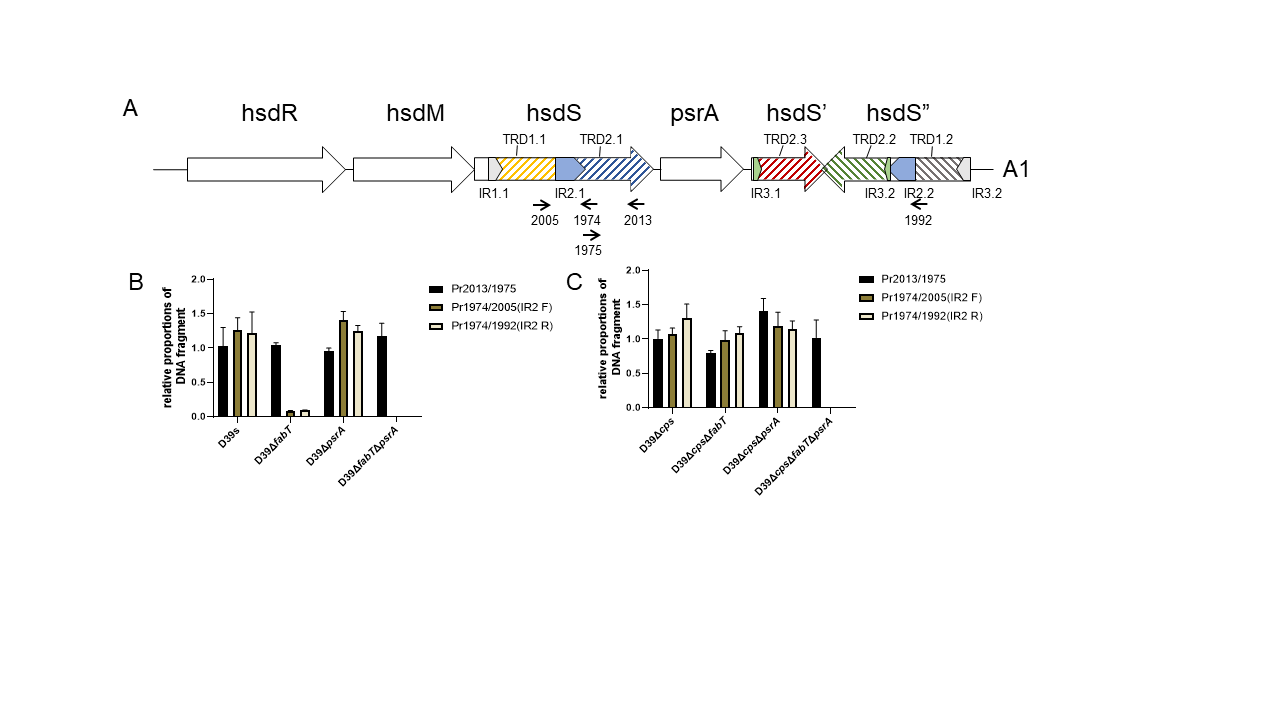

Supplement: FIG S5 [file mbio.01304-21-sf005.tif]

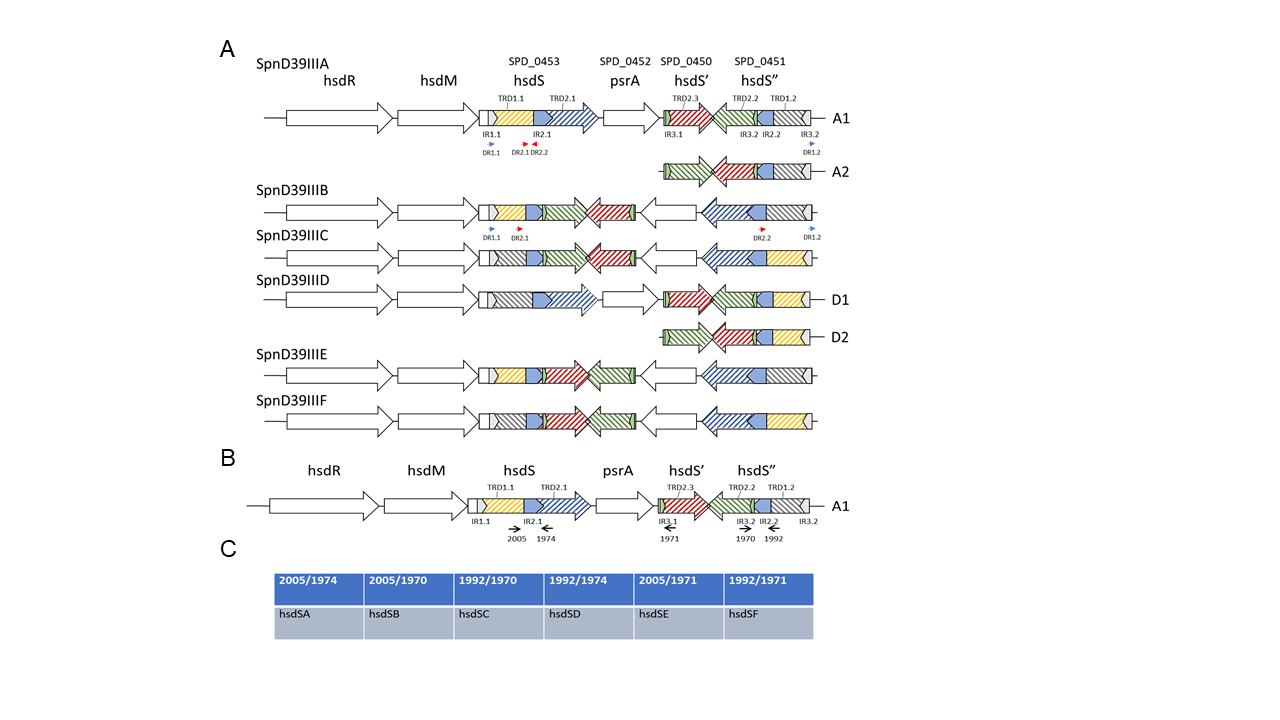

Supplement: FIG S6 [file mbio.01304-21-sf006.tif]
