# Supplementary material for: Inactivation of Transcriptional Regulator FabT Influences Colony Phase Variation of Streptococcus pneumoniae
Source: mBio. 2021 Aug 17;12(4):e01304-21. doi: 10.1128/mBio.01304-21 (PMC8406281; doi:10.1128/mBio.01304-21)
Supplement: TABLE S1 [file mbio.01304-21-st001.pdf]

Table S1 Quantitative assessment of the stability in the colony opacity phenotypes among the D39s derivatives

Strain (genotype)	Opacity phenotype of seeding colony	No. of colonies		Percentage of the opaque*
		Opaque	Transparent	
D39s	Opaque	100	0	100
		100	0	
		100	0	
D39 Δ*fabT*	Opaque	28	72	19±8
		18	82	
		12	88	
	Transparent	23	77	15±7
		12	88	
		10	90	
D39C Δ*fabT*	Opaque	100	0	100
		100	0	
		100	0	
	Transparent	0	100	0
		0	100	
		0	100	
D39 Δ*cps*	Opaque	100	0	100
		100	0	
		100	1	
D39 Δ*cps* Δ*fabT*	Opaque	40	160	18±3
		42	158	
		31	169	
	Transparent	28	178	17±5
		45	155	
		29	171	
D39 Δ*cps* C Δ*fabT*	Opaque	100	0	100
		98	2	
		100	0	

* The averages ± standard deviations were calculated on the basis of the three duplicate plates.
