# Supplementary material for: Inactivation of Transcriptional Regulator FabT Influences Colony Phase Variation of Streptococcus pneumoniae
Source: mBio. 2021 Aug 17;12(4):e01304-21. doi: 10.1128/mBio.01304-21 (PMC8406281; doi:10.1128/mBio.01304-21)
Supplement: TABLE S2 [file mbio.01304-21-st002.pdf]

TABLE S2 . Bacterial strains and plasmids used in this study

Strain or plasmid	Description	Reference(s) or source	Primers
**Strains**			
D39	*S. pneumoniae* D39 strain, Capsulated strain serotype 2	NCTC	
D39s	D39; *rpsL::rpsL41*	This study	Pr1801-1804
D39Δ*fabT* ::JC1	D39s derivative; the *fabT* region is replaced with JC1	This study	Pr1910-1914, 1917
D39Δ*fabT*	D39Δ*fabT* ::JC1 derivative; the 1-381 region of *fabT* is removed	This study	
D39CΔ*fabT*	D39ΔfabT derivate, pPEPZ-Plac-RBS-fabT-FLAG3	This study	
D39Δ*psrA*	D39s derivative; p.His7ThrfsTer15	(27)	Pr1975,1994,2008-2013
D39Δ*fabT* Δ*psrA*	D39Δ*fabT* derivate; p.His7ThrfsTer15	This study	
D39Δ*dexB-cps2A* ::JC1	D39s derivative, the *dexB-cps2A* region is replaced with JC1	(38)	Pr1901-1906
D39Δ*cps*	D39Δ*dexB-cps2A* ::JC1 derivative; the entire *dexB-cps2A* region is removed	This study	Pr1907-1908
D39Δ*cps* Δ*fabT*	D39Δ*cps* derivative; the 1-381 region of *fabT* is removed	This study	
D39Δ*cps* CΔ*fabT*	D39ΔcpsΔfabT derivative; pPEPZ-Plac-RBS-fabT-FLAG3	This study	
D39Δ*cps* Δ*psrA*	D39Δ*cps* derivative; ΔpsrA His7ThrfsTer15	This study	
D39Δ*cps* Δ*fabT* Δ*psrA*	D39Δ*cps* Δ*fabT* derivative; ΔpsrA His7ThrfsTer15	This study	
**Plasmid**			
pPEPZ-P_{lac}	Integrative plasmid with IPTG inducible promoter	(48)	
pPEPZ-Pl_{ac}-RBS-*fabT* -FLAG3	Integrative plasmid with IPTG inducible promoter and Flag-tagged fabT	This study	Pr2043/2034, 2035/2044
