# Supplementary material for: Inactivation of Transcriptional Regulator FabT Influences Colony Phase Variation of Streptococcus pneumoniae
Source: mBio. 2021 Aug 17;12(4):e01304-21. doi: 10.1128/mBio.01304-21 (PMC8406281; doi:10.1128/mBio.01304-21)
Supplement: TABLE S3 [file mbio.01304-21-st003.pdf]

Table S3 Primers used in this work.

Primer ID	Oligonucleotide sequence (5'-3')	Discription
Pr1801	GAGTGTACAGGGACGTGCTGAC	rpsL41- P1
Pr1802	GGGCTGAGTTAGGTTTTGTAGGTGTC	rpsL41- P2
Pr1803	GACACCTACAAAACCTAACTCAGCCC	rpsL41- P3
Pr1804	GAAGCAGCAGTACCACGTTTACC	rpsL41- P4
Pr1805	GTTCGTATGCGTCCAGGGAT	gyrB F
Pr1806	ATACCACGCCCATCATCCAC	gyrB R
Pr1807	AGGCTCCAGATGTGACTCCA	fabT F
Pr1808	ACCACACGACGATCCTGTTC	fabT R
Pr1809	GTGCAGCCAGTATCCCGATT	fabH F
Pr1810	GTAAGAATGAGCGTGCCCCA	fabH R
Pr1811	GTCCTGAGGTCGCAAATGGT	fabM F
Pr1812	GCCCGCTTCATCTCTACCAA	fabM R
Pr1901	TTCCTGACGAGAAGGTAGTCAATAA	dexB up F
Pr1902	AAAGCATAAGGAAAGGGGCCTTATAGTAATTCCACACAGA	dexB up R
Pr1903	GGAGTTTTCAGCATTATCCTATAGGTGTTAATCATGAGTA	cps2A dw F
Pr1904	GTCTAGATGGACATTCCCTACTGGG	cps2A dw R
Pr1905	TCTGTGTGGAATTACTATAAGGCCCCTTTCCTTATGCTTT	Δcps-Janus Cassette F
Pr1906	TACTCATGATTAACACCTATAGGATAATGCTGAAAACTCC	Δcps-Janus Cassette R
Pr1907	ACTCATGATTAACACCTATACAAAAAGCACCTCAAAAAGGTATTACC	Δcps R
Pr1908	CCTTTTTGAGGTGCTTTTTGTATAGGTGTTAATCATGAGT	Δcps F
Pr1910	GGAGTTTTCAGCATTATCCTTTTCATATCCCTCCTTC	fabT up R
Pr1911	GAAGGAGGGATATGAAAAGGATAATGCTGAAAACTCC	ΔfabT-Janus Cassette F
Pr1912	GTCAAGCCTTTACCCATGACCCTTTCCTTATGCTTTTGG	ΔfabT-Janus Cassette R
Pr1913	CCAAAAGCATAAGGAAAGGGTCATGGGTAAAGGCTTGAC	fabT dw F
Pr1914	CTGCAAGCCGCGGTTAAGTC	fabT dw R
Pr1915	GTCAAGCCTTTACCCATGACTTTTCATATCCCTCCTTC	ΔfabT R
Pr1916	GAAGGAGGGATATGAAAAGTCATGGGTAAAGGCTTGAC	ΔfabT F
Pr1917	GAGGTTGCAGATGGAAGCTG	fabT up F
Pr1930	GTTCACTTACACGAGCCCCA	psrA F
Pr1931	TGTCAAAGTACAGCAAAAACCGT	psrA R
Pr1995	GAATTATCAAGCGGAAAGAGCAAC	5’ non-invertible region in hsdS F
Pr1970	TTCATATATAGGATAAGAGGTTGTTTCGTCTTTG	IR2.2-2.1 R
Pr1971	AAATATCTTTTATTTTTATAACAACCCAATTCATAGGTATA	IR3.2-3.1 R
Pr1974	TCCCAACTTTCTGGTATTTCACAAGGTAC	IR2.1-2.2 R
Pr1975	GTACCTTGTGAAATACCAGAAAGTTGGGA	ΔpsrA::JC dw R
Pr1991	GTTAATTGGTAAAAAATAACAGGTGGTCAAACTG	IR3 F
Pr1992	GTGGAGCTGTTGTGAAAAACTTGAATAG	IR1.2-1.1 F
Pr1994	CCATAATCACAAAAATAGCAGGTAGTCAG	ΔpsrA::JC up F
Pr2001	CCACGAAATATCTCAGTTTCC	5’ non-invertible region in hsdS R
Pr2002	CTTATCAGTGAAGGAAAAATCAAACG	IR1 F
Pr2003	GTAAAGTATCCAAAACAATAAATGCTG	IR1R R

Pr2004	CATCCATCGTGTATTGCACCATCAAC	IR1F R
Pr2005	AAAGTTGCTTCTATTCTTATCCCTCTCCC	IR1.1-1.2 F
Pr2006	TGAGTGGCAGGAATATCCAATATGGC	IR3R R
Pr2007	ATCCACCAGCCACAACACCATCATAG	IR3F R
Pr2008	GCTTTCTTCAAATGTTAATTCAAAATC	ΔpsrA F
Pr2009	GATTTTGAATTAACATTTGAAGAAAGC	ΔpsrA R
Pr2010	AGGAGTTTTCAGCATTATCCTCTAGAGTCAATTTGCGACTACAAGG	ΔpsrA::JC up R
Pr2011	CCTTGTAGTCGCAAATTGACTCTAGAGGATAATGCTGAAAACTCCT	ΔpsrA-Janus Cassette F
Pr2012	CGTGCGGTGGAATTTCTATCCTCGAGCCTTTCCTTATGCTTTTGGAC	ΔpsrA-Janus Cassette R
Pr2013	GTCCAAAAGCATAAGGAAAGGCTCGAGGATAGAAATTCCACCGCACG	ΔpsrA::JC dw F
Pr2034	TTATTTATCATCATCATCTTTATAATCTTTATCATCATCATCTTTATAAGGTTCCGCTGGCTCCGCTGCTGGTTCTGGCGATTATAAAGATGATG	
Pr2035	ATGATAAAGATTATAAAGATGATGATGATAAAGATTATAAAGATGATGATGATAAATAATGGCTTTTGCAAAAATAAGT	Construction of pPEPZ-Plac-RBS-fabT-FLAG3
Pr2043	TTGAGATCTGAAGAAGGAGGGATATGAAAA	
Pr2044	CCGCTCGAGAAAAGCCATTATTTATCATC	
Pr2062	GCGTCTTGTTACGCCAAGTC	hsdM F
Pr2063	TCTCCTGAGTTCCCAGCATT	hsdM R
Pr2066	ACTCCTTTGAACCTTCGGCA	adcAII F
Pr2067	CATCCCCTGCTTCCACATCC	adcAII R
